# Women’s perceptions and attitudes towards the use of AI in mammography in Sweden: a qualitative interview study

**DOI:** 10.1136/bmjopen-2024-084014

**Published:** 2024-02-14

**Authors:** Jennifer Viberg Johansson, Karin Dembrower, Fredrik Strand, Åsa Grauman

**Affiliations:** 1 Centre for Research Ethics & Bioethics (CRB), Department of Public Health and Caring Sciences, Uppsala University, Uppsala, Sweden; 2 Capio S:t Görans Hospital, Stockholm, Sweden; 3 Department of Oncology-Pathology, Karolinska Institute, Stockholm, Sweden

**Keywords:** radiology & imaging, patient participation, patient satisfaction

## Abstract

**Background:**

Understanding women’s perspectives can help to create an effective and acceptable artificial intelligence (AI) implementation for triaging mammograms, ensuring a high proportion of screening-detected cancer. This study aimed to explore Swedish women’s perceptions and attitudes towards the use of AI in mammography.

**Method:**

Semistructured interviews were conducted with 16 women recruited in the spring of 2023 at Capio S:t Görans Hospital, Sweden, during an ongoing clinical trial of AI in screening (ScreenTrustCAD, NCT 04778670) with Philips equipment. The interview transcripts were analysed using inductive thematic content analysis.

**Results:**

In general, women viewed AI as an excellent complementary tool to help radiologists in their decision-making, rather than a complete replacement of their expertise. To trust the AI, the women requested a thorough evaluation, transparency about AI usage in healthcare, and the involvement of a radiologist in the assessment. They would rather be more worried because of being called in more often for scans than risk having overlooked a sign of cancer. They expressed substantial trust in the healthcare system if the implementation of AI was to become a standard practice.

**Conclusion:**

The findings suggest that the interviewed women, in general, hold a positive attitude towards the implementation of AI in mammography; nonetheless, they expect and demand more from an AI than a radiologist. Effective communication regarding the role and limitations of AI is crucial to ensure that patients understand the purpose and potential outcomes of AI-assisted healthcare.

**STRENGTHS AND LIMITATIONS OF THIS STUDY**

- The study’s strength lies in interviewing women actively undergoing mammography with artificial intelligence (AI) as a third reviewer, addressing hypothetical bias.
- This study provides valuable input for future ethical and legal considerations in AI integration in healthcare.
- Findings may be specific to the AI tool at Capio S:t Görans Hospital with Philips equipment, limiting transferability to other AI tools.
- The study primarily included participants with high trust in healthcare, potentially biasing results towards positive attitudes about AI implementation.

## Introduction

Population-based breast cancer screening programmes face the challenge of a heavy workload for radiologists assessing the breast X-ray images of mostly healthy women. Another challenge is the proportion of clinically detected breast cancers during the time between two consecutive screening examinations. These cancers, known as interval cancers, are associated with increased rates of both mortality and morbidity.^1^ There are several ways in which artificial intelligence software (AI) could improve the screening process. An AI cancer detection algorithm could work as a concurrent assistant to a radiologist and find additional cancers. It can also be used as an independent reader of the mammograms to reduce the workload for the radiologists as well as help in triaging patients in first-line care.^2^

AI systems have been introduced in some hospitals and clinics in Sweden, and their use is expected to increase dramatically in the coming years.^3 4^ Retrospective studies have demonstrated that an AI cancer detection algorithm could perform on par with, or even better than, radiologists.^2^ In recent years, there has been great progress in terms of improving AI accuracy as well as evaluating it as a tool, and several tumour detection algorithms have been developed for mammography.^5–7^ Retrospective studies have shown that standalone AI could assess mammograms with equal accuracy as radiologists.^8^ Two recent prospective clinical studies have confirmed that AI can be integrated into the screening workflow and help radiologists detect more cases of cancer at a lower cost.^9 10^ However, challenges such as overdiagnosis remain, which should be considered in further development and implementation.^11^

Dembrower *et al* 9 performed a prospective clinical trial following a paired screen-positive study design. In total, 55 581 women were included between April 2021 and June 2022. The aim of that study was to assess whether one reader in a double reading setting for screening mammograms could be replaced by an AI algorithm (AI tool with Philips equipment). The study determined that such an algorithm led to increased cancer detection and a lower recall rate than standard-of-care double reading by two radiologists. We have built on this study to explore women’s perceptions, based on the idea that their direct experiences in this specific context yield more authentic insights.

A high attendance rate is important for population-based screening programmes to reach more women. The breast screening programme in Sweden has high participation, with approximately 75%. High participation rates are important, but also crucial is ensuring this comes from women’s informed choices and trust in the AI’s reliability and the programme’s integrity. Therefore, women attending the screening programme are important stakeholders in the implementation of AI. To ensure women’s trust in the screening programme and survival rates, it is crucial to address practical aspects when considering the implementation of AI systems in medical imaging.^12^ Understanding women’s views is essential for an AI implementation that is not only efficient but also respectful of their informed decision-making rights.

Assessing the attitudes of the public, patients and healthcare personnel towards the use of AI in healthcare is a topic of great interest.^13^ Studies suggest a positive outlook on AI in healthcare, yet emphasise the need for human involvement in decision-making, highlighting the importance of a balanced, respectful approach^14^ and appreciate human interaction in the diagnostic process.^15–17^ Some studies also indicate that women want to be fully informed about the use of AI in mammography.^18^

This study aimed to explore perceptions and attitudes towards the use of AI in the national Swedish breast cancer screening programme among women participating in the national breast cancer screening programme.

## Methods

This article is a component of the AICare research project, which seeks to identify and anticipate legal challenges, while also proposing measures to ensure patient safety and enhance public acceptance of AI in healthcare. Conducted as a qualitative, semistructured interview study, this article will contribute to informing future legal and ethical analyses in this field.

### The setting and recruitment

Every day, 350 women, between 40 and 74, undergo their biannual screening examination at the hospital. Participation in the screening programme is voluntary and free of charge. The participants can reschedule their appointment online or via telephone if necessary. On arrival, the participants register their presence using their social security numbers and proceed to the waiting area where they await their turn. Subsequently, radiographers help the women throughout the screening examination, which generates four images, with two views of each breast. They also ask the women questions about clinical symptoms from their breasts, such as new lump(s), secretion, eczema and skin retraction. The screening examination lasts approximately 5 min. If there are no clinical findings and the mammograms are assessed with no suspicion of cancer, the women receive their mammography results by mail after approximately 2 weeks.

In the prospective clinical study, ScreenTrustCAD, the results of adding an AI algorithm with Philips equipment to the mammography reading at Capio S:t Göran hospital, were studied. If any reader, AI or radiologists flagged an examination for a suspicious image finding, the examination was discussed at a special meeting called a consensus discussion. On this occasion, at least two experienced breast radiologists assessed the mammograms and together decided whether the woman should be declared healthy or recalled for further follow-up. The current interview-based study was conducted at the same time as the ScreenTrustCAD study, that is, AI was already being used and informed for the women in this study.

Participants (ie, women participating in mammogram screening) were recruited at the mammography department at Capio S:t Göran Hospital, Sweden. In total, 63 women were invited by the first author (JVJ) to participate in the study before or after the screening procedure. All women were informed in the waiting room and in the consultation room that they were participating in a clinical study. Sixteen women consented, and two of them agreed to attend an interview via Zoom due to a lack of time in conjunction with their screening appointment.

### The participants

The participants were very positive about the breast cancer screening programme. The majority of the women had never been recalled. Those women who had reported a lump or been recalled expressed that it was very stressful. None of the women had been previously diagnosed and treated for breast cancer. Additional characteristics of the participants are presented in table 1.

**Table 1:** The characteristics of the participants (N=16) are presented as frequencies and percentages

Questions	
	n (%)
What is your age?	
41–50	5 (31.3)
51–60	6 (37.5)
61–70	5 (31.3)
70+	0 (0)
What is your highest level of education?	
Less than secondary/high school	1 (6.3)
Secondary/high school	4 (25.0)
University	11 (68.8)
Have you been called back for a follow-up of your mammogram?	
Never	13 (81.3)
Once	3 (18.8)
Compared with other individuals in Sweden of the same age, how do you assess your risk of developing breast cancer in the next 10 years?	
Slightly lower	1 (6.3)
Neither higher nor lower risk	7 (43.8)
Slightly higher	1 (6.3)
Much higher risk	2 (12.5)
Do not know/have no idea about it	5 (31.3)
Are you worried about developing breast cancer?	
Not worried at all	3 (18.8)
A little worried	12 (75.0)
Very worried	1 (6.3)
Has anyone in your immediate family been affected by breast cancer? (grandmother, aunt, mother or sister)	
Yes	2 (12.5)
No	12 (75.0)
Do not know	2 (12.5)

### Data collection

The interviews were performed in February 2023 by the first author (JVJ). They lasted from 15 to 36 min each, and they were conducted in Swedish, except one that was conducted in English. We began every interview by asking the participant about her experience of undergoing mammography. We introduced AI in a general context, inquiring about its capabilities. Thereafter, we delineated its application in mammography, illustrating how the AI, a computer program, scans mammogram images and detects irregularities, prompting a response if any deviation from healthy mammograms is detected. Thereafter, we asked about the women’s knowledge, thoughts, perceptions and attitudes of the technology in general, especially for evaluating mammograms. In this study, ‘perception’ refers to participants’ individual understanding and viewpoints on AI in breast screening, while ‘attitude’ encompasses their emotional and evaluative responses towards its use. See box 1 for the semistructured interview guide with open-ended questions.^19 20^

Box 1 **The interview guide used for the semistructured interviews primarily focused on open-ended questions to explore the women’s perceptions of artificial intelligence (AI) in evaluating their mammograms, although some follow-up questions were more direct to encourage deeper discussion and insight**

**Can you start by telling me about your experience undergoing mammography screening?**

**Description of how the mammogram images are assessed and the clinical trial with AI.**

- What do you think about that? How does it feel? To what extent do you perceive the assessment process as reliable?
- What do you think about AI in the assessment? What emotions does this information evoke?
- Do you feel okay with a doctor being replaced by AI? Could the involvement of AI change your trust in the assessment process? In what way?
- If we imagine that AI would replace both radiologists in the assessment, then you will not receive any other assessment by a doctor. Do you think that is okay (ie, no human involvement)?

**Description of AI and radiologist’s work and missing cancer.**

- What do you think about this? How do you feel about this?
- Would it be different if a doctor or AI misses seeing signs of cancer in the images? If the AI misses it, whose responsibility do you think it is? How do you feel about this?
- It is possible to set AI to signal for everything, even the smallest change, which would make doctors assess many images to determine if a woman should be contacted. As a result, many women will receive a letter stating that they need further tests to rule out cancer, even though not all women will have cancer. What do you think about that? Does it worry you?
- If AI is set to be less sensitive, fewer women will receive such a letter, reducing unnecessary worry. However, this would increase the risk of some women having undetected cancer. On the other hand, healthcare resources would be focused on more severe cases of cancer. What do you think about that? Does it worry you?
- In the long run, imagine AI replacing both radiologists to assess the images. What do you think about that? What risks or concerns do you have?

**Description of the development of healthcare through the sharing of healthcare data.**

- What information about yourself do you consider sensitive? What is acceptable for you to share within healthcare, and what is more sensitive?
- Is a mammogram image (show the image) sensitive if it were to be shared with other hospitals or companies developing these algorithms?
- How do you feel if no other information about you could be linked to your image, only the result of the examination (cancer or not)? Would that be sensitive for you?

**Closing questions**

- After discussing your perspective on using AI in mammography screening, do you have any final thoughts?
- Is there anything else we haven’t talked about that you would like to mention?

### Analysis

The recorded interviews were transcribed verbatim by a professional transcription company. Subsequently, the audio files were listened to in their entirety to verify the transcriptions. After all the transcripts were read again, meaning units (phrases, sentences or paragraphs) related to the aim were identified for further scrutiny. The data management and analysis process was facilitated by using Atlas.ti Web^21^ Microsoft Excel (2016) as a tool. The next phase of the process entailed comparisons of the meaning units, examining their similarities and differences from the perspective of perception of AI in mammography. Open coding of each meaning unit was added. Two of the interviews were coded simultaneously by both authors (JVJ and ÅG), who jointly discussed what meaning units to identify, interpretations and formulations of codes; they also created coding frameworks to improve conceptual clarity or transparency in the data analysis. The remaining interviews were coded by JVJ, but regular follow-up meetings were held to discuss new codes. Codes that reflected a similar concept were grouped; subcategories were formulated, and categories were identified^22 23^ by JVJ and thereafter discussed thoroughly with the other authors (ÅG, FS and KD) (see table 2). Thematic saturation was attained in alignment with the intended objectives of the data collection.^24^

**Table 2:** An example of the analytical process regarding the perception of using AI as a decision tool evolved from identifying ‘meaning units’ and performing ‘initial coding’ to organising codes into ‘subcategories’ and, ultimately, formulating overarching ‘categories’ for a more comprehensive qualitative analysis

Meaning unit	Initial coding	Subcategory	Category
*‘I think that a human being can… can see, like, ‘Oh, is there something there, then maybe I have to look there, and then I have to look there, and then I have to look there.’ Some kind of reasoning ability.’*	Humans can see connections; it’s like detective work	AI is merely a tool	Perceived differences between AI and human assessment
*‘If it is used as a complement, I think it is good.’*	AI should be used as a complement	Human in the loop	Requirements when using AI in mammography
*‘…anonymization is important, ensuring that the AI company or any other party does not have access to personal data or information about individuals. It’s crucial to protect privacy and prevent sensitive data from being linked to specific individuals.’*	Consider sharing deidentified mammography images with AI companies	Willingness to data sharing varies in routine care, research and industry	Attitudes when implementing AI in mammography

AI, artificial intelligence.

### Patient and public involvement

None.

## Results

The results below describe the perceptions and attitudes expressed by the participants regarding AI in healthcare. We identified 318 meaning units in total. When analysing them, seven subcategories emerged, which were categorised into three main categories: perceived differences between AI and human assessment, trust when implementing AI in mammography and requirements when using AI in mammography. An overview of these subcategories and main categories is presented in table 3. These will be described and illustrated using quotes in the following sections.

**Table 3:** The categories and subcategories regarding participants’ perceptions of taking part in triage for mammography using AI

Category	Subcategory
Perceived differences between AI and human assessment	AI is merely a tool
	The difference in error tolerance
Attitudes when implementing AI in mammography	Trust in the healthcare system gives confidence in AI
	Willingness to data sharing varies in routine care, research and industry
Requirements when using AI in mammography	Evaluation of AI
	Human in the loop
	The trade-off between being worried about possible cancer and detecting cancer

AI, artificial intelligence.

### Category: perceived differences between AI and human assessment

Within this category, the participants talked about the positive and negative characteristics of AI and human assessment. They also talked about how their moral expectations differed between AI and humans regarding error tolerance.

#### Subcategory: AI is merely a tool

The participants were familiar with AI to varying degrees, primarily through their interactions with search engines and recommendation systems on the internet. In addition to the positive aspects, they had heard stories about AI being used to cheat when writing papers and exams. Some participants expressed excitement about the development of AI, recognising its potential benefits. However, one participant cautioned against the excessive hype, stating that AI should not be equated with the futuristic concept portrayed by some.

Despite the generally positive sentiment towards the utilisation of AI in mammography, a subset of participants voiced reservations regarding their limited understanding of the underlying mechanisms of AI. They expressed a sense of ambivalence towards forming a definitive opinion on the benefits or drawbacks of AI implementation in mammography. After a brief presentation on how AI works at the clinic, they were all capable of expressing their attitudes towards AI. They made clear distinctions between the characteristics and abilities of an AI compared with a human; they often drew on what humans are capable of when describing what AI can or cannot do.

No, but I mean, can you build a machine that is that skilled? That’s what I’m thinking. In addition, the human eye and experience of how things can look; I don’t think you can just cram all of that into a robot, you know. (Participant 7)

The women acknowledged and appreciated that AI has the potential to identify patterns that may elude the human eye and respond to deviations from the norm. They perceived that AI could be a powerful tool, helping radiologists complement their work. Some expected that the use of AI would save resources for the healthcare system and give radiologists the possibility of performing other tasks. Some perceived that the AI to a great extent would complement the radiologists. They believed that AI is self-learning, quick and will never get tired. Some participants held the belief that AI technology will continue to evolve over time, surpassing its current capabilities and becoming even more advanced. They expressed that the utilisation of AI represents the future of healthcare. Consequently, humans and AI were perceived as complementary entities, working together synergistically.

Nevertheless, a prevailing scepticism regarding the current capabilities of AI was evident among many participants. One expressed a general cautious perspective towards technology, acknowledging its benefits as long as it functions properly. However, some raised concerns about the sustainability of AI effectiveness in the long run. Some participants expressed uncertainty regarding AI’s ability to detect all cancers, stating that AI might have difficulty with certain more uncommon conditions, such as breast implants or dense breast tissue. They also doubted whether AI could be good enough to replace the two radiologists currently assessing the mammography images. On the other hand, AI could be good at detecting things that the human eye cannot. However, participants expressed uncertainty regarding the specific areas in which AI excels beyond the capabilities of the human brain.

I also think that a human has the ability to… some form of consequential thinking. I mean, a reasoning ability that AI possibly doesn’t have. (Participant 6)

Some of the major strengths attributed to humans included their holistic perspective, considering various factors related to the individual woman, and making connections between different pieces of information. Humans were also perceived as capable of thinking about consequences, conducting investigative work and demonstrating greater imagination. Overall, human abilities were perceived in a more positive sense compared with AI. Many participants, however, expressed a willingness to accept the use of AI if it could outperform radiologists in terms of detecting more cases of cancer. The primary concern for them was the timely and accurate identification of cancer, regardless of the method used. However, they also strongly believed that human involvement adds an irreplaceable element that AI currently lacks and perhaps never will possess. Participants emphasised that humans bring unique qualities and abilities to the screening process that go beyond mere detection, such as intuition, empathy and contextual understanding. While they acknowledged the potential of AI, they maintained that human touch and contribution are invaluable and offer something distinct that AI cannot yet replicate.

I’m not a hundred percent convinced that the human eye can be replaced because there are… we also had scanning microscopes and things like that. That was a hundred years ago now… but, I mean, it [AI] is an aid. (Participant 3)

#### Subcategory: the difference in error tolerance

The ability of AI to detect cancer in mammography was consistently emphasised by all participants, and the failure to detect cancer was perceived as devastating. However, their acceptance of failure to detect cancer differed between radiologists and AIs, having a much higher acceptance for mistakes made by radiologists than by an AI. This was because participants felt that to err is human—and therefore understandable. In contrast, the majority expected that technology, including AI, should not make mistakes when used in healthcare. They believed that AI, to be acceptable on implementation, should function well, maintain a high level of security and operate without errors. They questioned the rationale behind implementing AI if it did not meet these criteria. Two participants specifically highlighted the potential consequences of AI-making mistakes that could be catastrophic due to the systematic nature of errors. In contrast, they pointed out that if a single radiologist were to miss a few cases of cancer in a day, the impact would be comparatively limited. The participants expressed a range of negative emotions, including feeling bad, disappointment and cheating if AI, were to make a mistake. One participant held a pessimistic view of technology and questioned whether it could guarantee reliable performance at all times, despite acknowledging its current functionality.

Yes, because we all know that a technology […] it should be a hundred percent… (Participant 2)

A few participants expressed a more nuanced perspective, acknowledging that neither humans nor AI can be expected to be 100% accurate all the time. They recognised that AI relies on data and statistical predictions, which inherently introduces the possibility of errors. These participants emphasised that no system, whether human or AI, is infallible, and both can potentially make mistakes in their assessments.

The participants held high expectations of the radiologists and regarded them as having ultimate responsibility for the final results. Additionally, some participants believed that if AI were to make a mistake, it would be the responsibility of humans, as AI is viewed as a tool incapable of being held accountable for its decisions.

[…] It is always the medically responsible physician, the radiologist, who is accountable. We can’t blame the technology. We must have technology that is tested and that we believe in and trust. It’s always the medically responsible physician, in my opinion. Where else should the responsibility lie, I wonder. That’s how I think. (Participant 4)

However, one respondent expressed an opposing viewpoint, suggesting that if a radiologist were to make a mistake, it could be attributed to a flaw in the device or image-computer system. Another participant highlighted the importance of the radiologist not disregarding the indications provided by AI when it signals a case of potential cancer. Overall, the participants’ perceptions varied regarding the issue of responsibility.

### Category: attitudes when implementing AI in mammography

This category and its subcategories concerned women’s attitudes in sharing mammography images with different actors, as well as trust in the healthcare system’s implementation of AI. Here, we identified two subcategories.

#### Subcategory: trust in the healthcare system gives confidence in AI

This subcategory predominantly reflects the participants’ strong trust in the healthcare system in regard to implementing AI in routine practices. All the participants expressed a high level of confidence that healthcare providers would take the necessary steps to deliver the best possible care, even if it involved the implementation of AI.

I trust that hospitals do it to the best of their ability. (Participant 6)

While some participants acknowledged the potential for unwanted motivations, such as cost-saving measures behind AI implementation, they still expressed trust that both healthcare providers and developers of AI tools have the intention of saving lives and improving patient outcomes. Some participants even indicated that if healthcare professionals have good intentions in using AI, they do not feel the need to have detailed information regarding its use.

No, I think experts must have examined and evaluated that, so no, I would probably trust… I mean, I think if they have chosen it, it is because it’s very effective and accurate. (Participant 9)

#### Subcategory: willingness to data sharing varies in routine care, research and industry

The participants consistently expressed their willingness to share their health information within the healthcare system setting. Furthermore, many participants held a strong belief that different healthcare units should collaborate and share information more extensively than they currently do, emphasising that such sharing is crucial to ensure patient safety. However, in regard to sharing health-related data or personal data with companies, the participants were more reluctant. For some, it was perceived as a great violation of their privacy. The participants felt that mammogram images of their breasts were less controversial if they were completely anonymised and not connected to them as individuals. Anonymisation of the mammograms helped to alleviate concerns regarding privacy and personal identification.

No, I wouldn’t have any issues with that [sharing mammograms] as long as it can’t be linked to me as a specific individual. (Participant 6)

However, for many of the participants, providing consent to share the data was viewed as necessary and connected to their ability to influence that the mammograms would be used for a meaningful purpose.

On the other hand, if it’s a research project or a pilot project, then you want to know that you’re participating in such a project. However, if it’s part of the established methodology at hospitals… I don’t interfere with that. (Participant 6)

I would like to know beforehand so that they don’t just send it off without informing me. It doesn’t feel entirely right. I would expect to be informed about it, though. (Participant 8)

They were willing to consent if the purpose was to save lives, develop new medical devices or conduct research. Conversely, if the data usage was intended for commercial companies or advertising purposes, they expressed a strong reluctance to share their data.

### Category: requirements when using AI in mammography

This category reflects the prerequisites expressed by our participants regarding the use of AI in the mammography screening process.

#### Subcategory: evaluation of AI

This subcategory related to the paramount evaluation of AI in multiple aspects. Participants highlighted the need to evaluate AI’s cancer detection performance compared with radiologists and the overall integration process and integration process. Participants stressed evaluating professionals’ perspectives, AI image selection criteria and the impact on radiologists’ skills. They underscored the importance of radiologists’ continuous training, decision-makers’ understanding of AI’s implications, and the need for control and monitoring to ensure AI’s accuracy and relevance to the Swedish context.

The participants expressed concern that decision-makers might focus solely on certain aspects, such as cost savings, when evaluating AI, potentially overlooking other important factors, such as providing comprehensive training for specialists.

The participants agreed that the evaluation of the AI’s performance itself needs to focus on the ability of AI to keep the margin of error down and evaluate to what extent it performs better.

I think it’s possible to evaluate the error margins and compare the percentage of correct or incorrect findings between the two. Therefore, I believe that would be beneficial. (Participant 5)

#### Subcategory: human in the loop

All participants preferred humans over AI; however, they expressed that combining AI and humans would be a complement to each other. The participants emphasised the need for human oversight due to their perception of the unique qualities possessed by radiologists and humans; even though AI is a powerful tool, it requires human control. They expressed the desire to harness the combined strengths of both humans and AI. Moreover, participants believed that a collaborative approach, where AI assists radiologists in detecting abnormalities, would yield the best outcomes.

No, but I think… as long as there is a human factor involved, I believe it can be very effective. (Participant 6)

Some of the participants identified the risk that using AI too much would lead to a decline in learning skill development for the radiologist. They reasoned that if AI gives too many false positives, it will create a misleading foundation for radiologists to identify cancer cases. They expressed concerns that if AI solely performed the initial assessment, radiologists would only discuss a selected sample of images identified by AI. One respondent had a different perspective and was less concerned about radiologists losing competence. Instead, she understood that the development of AI requires radiologists to continuously improve their skills. She recognised that to effectively contribute to the development and training of AI systems, radiologists must stay updated and enhance their expertise. This viewpoint highlights the symbiotic relationship between radiologists and AI, where ongoing skill development and collaboration contribute to the advancement of both fields.

But it is still the case that radiologists constantly need to enhance their competence to develop AI. Because it’s the people who collect the data; it’s not like an external force that does it. (Participant 4)

Some expressed a hopeful perspective regarding the combination of AI and human involvement in preventing potential errors. They perceived that there is too much uncertainty regarding the performance of AI, which led them to believe that relying solely on AI at the present moment would be a substantial leap. They expressed fears that something will be lost and that the radiologists will undermine their role in the diagnostic process; AI can serve as a support tool, but a human needs to be in control. All participants thought it was acceptable to implement AI as long as one radiologist was still involved in the first round. However, one participant expressed that it was compulsory to like digitisation, as it is a reflection of our society’s direction. She expressed a preference for a collaborative approach, desiring the presence of a radiologist in the process and appreciation for human contact and interaction in the process.

#### Subcategory: the trade-off between being worried about possible cancer and detecting cancer

The participants strongly expressed that failing to detect a case of cancer is terrible, and they emphasised the importance of healthcare providers doing everything possible to ensure early detection. They recognised the trade-off between causing increased worry among women due to more false positive cases and the potential risk of missing a cancer diagnosis after explaining that this was the reality. However, they expressed a clear preference for prioritising the detection of potential cancer cases over minimising false positives. They believed that it was better for more individuals to experience the anxiety of false-positive results than for a potential case of cancer to go undetected.

I think we should be a bit more… we should be able to live a bit more in reality [not being afraid of bad news]. And if we can get the help [hinder cancer] that it entails, that I have to go for an extra appointment, then I would just be thankful and accept it. I’m not inclined in the other direction. No, I would rather go for the extra appointment than not do it. (Participant 4)

Participants agreed on setting AI to a highly sensitive level for better cancer detection and emphasised the need for transparent communication about AI use in Swedish healthcare. They believed women would accept AI if it improved cancer detection and advocated for detailed information in referral letters about potential increased callbacks due to AI’s sensitivity.

Then, they must communicate that ‘the experience has shown that about thirty percent of those who have this develop cancer, or zero or twenty or forty.’ Something like that. I think it’s good to communicate that, if you ask me. (Participant 1)

Participants preferred immediate follow-ups to lessen anxiety, viewing screening as an act of solidarity and accepting false positives for timely cancer treatment for others. They saw the initial resource investment in AI as ultimately cost-saving through early detection. Emphasising transparency to maintain trust, they indicated they would not opt out of mammography with AI integration, underscoring the importance of open communication in healthcare AI.

## Discussion

### The main findings

The main findings of this study demonstrate that participants perceive AI as a valuable tool in mammography, recognising the potential benefits and its ability to complement the work performed by radiologists. However, there are concerns about participants’ limited understanding of AI and reservations about its current capabilities. Participants stressed human involvement, transparent screening and evaluating AI’s ability to detect cancer. They prioritised finding potential cancers over reducing false positives and believed that women would accept AI if it detected more cases. The emphasis on cancer detection, while crucial, poses challenges due to limited healthcare resources. Lowering sensitivity thresholds raises ethical questions about costs and personnel. It is imperative to explore the lessons learnt from Sweden’s mammography programme and consider the ethical, defensible trade-offs, especially in resource allocation. Striking a balance between optimal detection and efficient resource use remains a pivotal area for healthcare policy discussions.

The results underscore the importance of a collaborative approach, where AI and radiologists complement each other and where radiologists maintain a vital role in the diagnostic process by contributing with their ‘human capabilities’. These findings align with other patient groups, where participants emphasised the need for, and value of, information, even if it is not used in medical decision-making.^25^ The study shows that it is crucial to inform patients about the use of AI as a straightforward clinical implication, aiming to maintain participants’ trust. In addition, the study highlights that human involvement in medical care is highly valued, and it would be difficult to replace it with AI. This is a consistent theme identified in studies outside the realm of mammography.^26 27^

### Discussion of each finding

Within the first category in our study, we discovered perceptions regarding differences between AI and human assessment. Participants were cautiously optimistic about AI in healthcare, acknowledging its benefits but expressing reservations due to current limitations in detecting all cancers and conditions. Interestingly, these views align with those of participants in prior studies where concerns about AI replacing radiologists entirely, apprehensions regarding the absence of a ‘human touch’ in the diagnostic process,^14 17^ and accountability issues hindered the acceptance of AI-driven medicine.^28 29^ Moreover, our study emphasises the importance of retaining human expertise and clear lines of responsibility in breast cancer screening. Participants underlined these aspects, emphasising the need for a specific framework, although such guidelines are still under development. The National Board of Health and Welfare in Sweden has initiated work on guidelines for AI in healthcare,^30^ a step in the right direction. This emphasis aligns with prior research stressing the importance of preserving human involvement in breast screening.^31 32^ It highlights the complexity of AI perception in healthcare, emphasising the necessity for tailored guidelines that address regulatory and ethical concerns while catering to the specific demands of distinct medical fields.

It is particularly intriguing to note that participants in our study placed significant emphasis on the consequences of technological errors. Most women held high expectations for AI, anticipating its flawless performance, unwavering security and a complete absence of errors. Strikingly, when contemplating the prospect of AI making mistakes, participants expressed strong negative emotions. This contrasts with the perceptions of healthcare professionals, where a certain degree of error was deemed more acceptable, attributed to the inherent human factor.^33 34^ People in general tolerate fewer mistakes made by technical products than mistakes made by humans. A pivotal observation was the varying perspectives on responsibility among the participants. While many ascribed the ultimate responsibility for potential errors to radiologists, a subset viewed AI as an entity lacking in accountability. These attitudes describe the intricate interplay between human trust, the technology’s reliability and the attribution of responsibility in the context of AI implementation within healthcare. This divergence in tolerance towards errors by humans versus AI underscores a psychological aspect of trust in healthcare. Participants view human errors as part of learning, but see AI mistakes as systemic flaws, challenging trust in technology. This dichotomy raises critical questions about how we educate and prepare the public for the realistic capabilities and limitations of AI in healthcare, balancing technological optimism with a pragmatic understanding of AI as an evolving tool rather than an infallible solution.

In our study, the category ‘Attitudes when Implementing AI in Mammography’ revealed that participants generally exhibited trust in the integration of AI into routine mammography practices. They believed that healthcare providers and AI developers shared a common goal of saving lives and enhancing patient outcomes. However, willingness had boundaries, as participants were reluctant to share health information with external companies, considering it an intrusion into their private lives.

The participants expressed that maintaining the confidentiality of the mammogram images played a pivotal role in addressing privacy concerns, making participants more willing to share their data as long as it could not be traced back to them as individuals. Consent for data sharing was often contingent on meaningful purposes, such as life-saving measures or research initiatives. Conversely, participants expressed hesitation about sharing data for commercial or advertising purposes. This aligns with previous studies on people’s attitudes towards data sharing.^35^ There is a risk that the participants do not comprehend the necessity of sharing data with private entities to advance technology.

In summary, our study underscores the pivotal role of attitudes in AI implementation in healthcare. Trust, rooted in transparent collaboration between healthcare providers and participants, is fundamental. Recognising its importance is crucial in the complex realm of AI integration. Collaborative efforts, informed by human rights frameworks and practical recommendations, are essential. These actions not only preserve but also nurture trust, ensuring the inclusive and effective application of AI technology in healthcare.^36 37^

In the third category, participants expressed their thoughts on prerequisites for the implementation of AI in mammography screening. In line with these expressed prerequisites, it is evident that the implementation of AI in healthcare, particularly in mammography screening, has brought to the forefront a multitude of ethical considerations. These considerations closely align with the broader discussions in the literature surrounding the ethical dimensions of AI integration.^11 38^ These include evaluating AI’s performance compared with that of radiologists, ensuring transparency in the screening process and addressing concerns about the potential loss of expertise among radiologists. The participants stressed the importance of ongoing training for radiologists and urged decision-makers to consider factors beyond cost savings. It is, therefore, important that there is a solution for unexperienced radiologists to undergo training in mammography screening. Trust in AI is expected to increase as familiarity with its capabilities grows. This was also observed among the radiologists when the ScreenTrust CAD study started, as recall rates increased during the first few months and later normalised. Although participants in that study preferred human involvement in the screening process, they recognised the potential of AI in detecting abnormalities and believed that a combination of both would yield the best outcomes, which the ScreenTrust CAD study confirmed.^9^ Participants prioritised the early detection of cancer, even if it meant accepting more false-positive results and having to return for further follow-up examinations. Participants saw the implementation of AI as initially requiring additional resources but ultimately saving money by detecting cancers earlier with fewer resources.

### Strengths and limitations

This study, focusing on women’s perceptions and attitudes towards AI in mammography, offers insights for shaping ethical frameworks and legal guidelines in AI healthcare applications. While not a bioethical analysis per se, it provides valuable input for future ethical and legal considerations in AI integration in healthcare. However, the findings may only be applicable to this AI tool at Capio S:t Görans Hospital with Philips equipment. The study excluded other AI-developed tools, which may limit the transferability of the results. One major strength of our study is that we interviewed women who were actively undergoing mammography, where AI was used as a third reviewer. This means that we have addressed the hypothetical bias commonly encountered in studies.^39^

There are potential limitations in self-reporting bias and subjectivity in interview-based studies. To address these limitations, the researchers encouraged honest and candid responses, maintained confidentiality, reflected on their own perspectives and biases, sought input from colleagues and used coding frameworks to improve objectivity in data analysis.^40^ Considering transferability, we identify a limitation: our study predominantly intercepted participants who exhibited high trust in healthcare, a sentiment likely to influence their confidence in the implementation of AI. To address this gap, we recommend examining specific demographic groups to gain comprehensive insights. Some people with low trust in conventional healthcare might also be sceptical about AI, while others might perceive AI as more objective, leading to higher trust. This viewpoint is supported by Yang *et al*,^26^ indicating varied levels of trust based on demographics. Understanding these dynamics is vital to grasp societal attitudes towards AI in healthcare.

### Relevance

In summary, this study’s findings hold significant relevance for the ongoing debate surrounding AI in healthcare, especially within breast cancer screening programmes. This highlights the essential role of trust in AI and the necessity of retaining human involvement in making decisions regarding mammogram images. These insights are vital for ensuring the effective and well-received implementation of AI, emphasising transparent communication and comprehensive patient education regarding the role of AI in breast cancer screening.

In this study, participants clearly emphasised the ethical principles of fairness, privacy, responsibility and accuracy while underscoring the competence of radiologists and the importance of transparency. Their overall positive attitude towards the technology is evident.

### Conclusion

The findings of this study indicate that women who participate in mammography view AI as one of many tools in the healthcare system, not as a standalone solution. They acknowledge its potential but emphasise its role in complementing existing practices rather than replacing the radiologists entirely. However, if healthcare professionals determine that AI functions equally or even better than traditional screening processes, then the participants find the implementation of AI without explicit consent acceptable. Notably, women prefer AI to be sensitive in detecting potential cancer, even if it may lead to an increased level of unnecessary worry and fear. Nevertheless, effective communication regarding the role and limitations of AI is crucial to help patients understand its purpose and potential outcomes in healthcare and to maintain their trust.

## Supplementary Material

Reviewer comments

Author's manuscript

## Data Availability

No data are available.
