# Supplementary material for: Women’s perceptions and attitudes towards the use of AI in mammography in Sweden: a qualitative interview study
Source: BMJ Open. 2024 Feb 14;14(2):e084014. doi: 10.1136/bmjopen-2024-084014 (PMC10868248; doi:10.1136/bmjopen-2024-084014)
Supplement: Author's manuscript [file bmjopen-2024-084014.draft_revisions.pdf]

BMJ Open

BMJ Open is committed to open peer review. As part of this commitment we make the peer review history of every article we publish publicly available.

When an article is published we post the peer reviewers' comments and the authors' responses online. We also post the versions of the paper that were used during peer review. These are the versions that the peer review comments apply to.

The versions of the paper that follow are the versions that were submitted during the peer review process. They are not the versions of record or the final published versions. They should not be cited or distributed as the published version of this manuscript.

BMJ Open is an open access journal and the full, final, typeset and author-corrected version of record of the manuscript is available on our site with no access controls, subscription charges or pay-per-view fees (http://bmjopen.bmj.com).

If you have any questions on BMJ Open's open peer review process please email info.bmjopen@bmj.com

BMJ Open

Women's perceptions and attitudes toward the use of AI in mammography

Journal:	*BMJ Open*
Manuscript ID	bmjopen-2024-084014
Article Type:	Original research
Date Submitted by the Author:	05-Jan-2024

Complete List of Authors:	Viberg Johansson, Jennifer; Uppsala University Department of Public Health and Caring Sciences, Centre for Research Ethics & Bioethics (CRB)<br>Dembrower, Karin; Capio Saint Gorans Hospital<br>Strand, F; Karolinska Institute, Department of Oncology-Pathology<br>Grauman, Å; Uppsala Universitet, Department of Public Health and Caring Sciences

Keywords:	RADIOLOGY & IMAGING, Patient Participation, Patient Satisfaction

SCHOLARONE™ Manuscripts

BMJ

I, the Submitting Author has the right to grant and does grant on behalf of all authors of the Work (as defined in the below author licence), an exclusive licence and/or a non-exclusive licence for contributions from authors who are: i) UK Crown employees; ii) where BMJ has agreed a CC-BY licence shall apply, and/or iii) in accordance with the terms applicable for US Federal Government officers or employees acting as part of their official duties; on a worldwide, perpetual, irrevocable, royalty-free basis to BMJ Publishing Group Ltd ("BMJ") its licensees and where the relevant Journal is co-owned by BMJ to the co-owners of the Journal, to publish the Work in this journal and any other BMJ products and to exploit all rights, as set out in our licence.

The Submitting Author accepts and understands that any supply made under these terms is made by BMJ to the Submitting Author unless you are acting as an employee on behalf of your employer or a postgraduate student of an affiliated institution which is paying any applicable article publishing charge ("APC") for Open Access articles. Where the Submitting Author wishes to make the Work available on an Open Access basis (and intends to pay the relevant APC), the terms of reuse of such Open Access shall be governed by a Creative Commons licence – details of these licences and which Creative Commons licence will apply to this Work are set out in our licence referred to above.

Other than as permitted in any relevant BMJ Author's Self Archiving Policies, I confirm this Work has not been accepted for publication elsewhere, is not being considered for publication elsewhere and does not duplicate material already published. I confirm all authors consent to publication of this Work and authorise the granting of this licence.

For peer review only

Journals: BMJ Open

Title: Women's perceptions and attitudes toward the use of AI in mammography

Abstract

Background: Understanding women's perspectives can help to create an effective and acceptable AI implementation for triaging mammograms, ensuring a high proportion of screening-detected cancer. This study aimed to explore Swedish women's perceptions and attitudes toward the use of AI in mammography.

Method: Semi-structured interviews were conducted with sixteen women (N=16) recruited in the spring of 2023 at Capio S:t Görans Hospital, Sweden, during an ongoing clinical trial of AI in screening (ScreenTrustCAD, NCT 04778670) with Philips equipment. The interview transcripts were analyzed using inductive thematic content analysis.

Results: In general, women viewed AI as an excellent complementary tool to help radiologists in their decision-making, rather than a complete replacement of their expertise. To trust the AI, the women requested a thorough evaluation, transparency about AI usage in healthcare, and the involvement of a radiologist in the assessment. They would rather be more worried because of being called in more often for scans than risk having overlooked a sign of cancer. They expressed substantial trust in the healthcare system if the implementation of AI was to become a standard practice.

Conclusion: The findings suggest that the interviewed women, in general, hold a positive attitude toward the implementation of AI in mammography; nonetheless, they expect and demand more from an AI than a radiologist. Effective communication regarding the role and limitations of AI is crucial to ensure that patients understand the purpose and potential outcomes of AI-assisted healthcare.

For peer review only

Introduction

Population-based breast cancer screening programs face the challenge of a heavy workload for radiologists assessing the breast X-ray images of mostly healthy women. Another challenge is the proportion of clinically detected breast cancers during the time between two consecutive screening examinations. These cancers, known as interval cancers, are associated with increased rates of both mortality and morbidity [1]. There are several ways in which artificial intelligence software (AI) could improve the screening process. An AI cancer detection algorithm could work as a concurrent assistant to a radiologist and find additional cancers. It can also be used as an independent reader of the mammograms to reduce the workload for the radiologists as well as help in triaging patients in first-line care [2]. AI systems have been introduced in some hospitals and clinics in Sweden, and their use is expected to increase dramatically in the coming years [3, 4]. Retrospective studies have demonstrated that an AI cancer detection algorithm could perform on par with, or even better than, radiologists [2]. In recent years, there has been great progress in terms of improving AI accuracy as well as evaluating it as a tool, and several tumor detection algorithms have been developed for mammography [5-7]. Retrospective studies have shown that standalone AI could assess mammograms with equal accuracy as radiologists [8]. Two recent prospective clinical studies have confirmed that AI can be integrated into the screening workflow and help radiologists detect more cases of cancer at a lower cost [9, 10]. However, challenges such as overdiagnosis remain, which should be considered in further development and implementation [11].

Dembrower [9] performed a prospective clinical trial following a paired screen-positive study design. In total, 55,581 women were included between April 2021 and June 2022. The aim of that study was to assess whether one reader in a double reading setting for screening mammograms could be replaced by an AI algorithm (AI tool with Philips equipment). The

For peer review only - http://bmjopen.bmj.com/site/about/guidelines.xhtml

52 study determined that such an algorithm led to increased cancer detection and a lower recall
53 rate than standard-of-care double reading by two radiologists. We've built upon this study to
54 explore women's perceptions, based on the idea that their direct experiences in this specific
55 context yield more authentic insights.

56 A high attendance rate is important for population-based screening programs to reach more
57 women. The breast screening program in Sweden has high participation, with approximately
58 75%. High participation rates are important, but also crucial is ensuring this comes from
59 women's informed choices and trust in the AI's reliability and the program's integrity.
60 Therefore, women attending the screening program are important stakeholders in the
61 implementation of AI. To ensure women's trust in the screening program and survival rates,
62 it is crucial to address practical aspects when considering the implementation of AI systems
63 in medical imaging [12]. Understanding women's views is essential for an AI implementation
64 that is not only efficient but also respectful of their informed decision-making rights.

65 Assessing the attitudes of the public, patients, and healthcare personnel toward the use of AI
66 in healthcare is a topic of great interest [13]. Studies suggest a positive outlook on AI in
67 healthcare, yet emphasize the need for human involvement in decision-making, highlighting
68 the importance of a balanced, respectful approach [14], and appreciate human interaction in
69 the diagnostic process [15, 16] [17]. Some studies also indicate that women want to be fully
70 informed about the use of AI in mammography [18].

71 This study aimed to explore perceptions and attitudes toward the use of AI in the national
72 Swedish breast cancer screening program among women participating in the national breast
73 cancer screening program.

74

For peer review only

Methods

This prospective study was approved by the Swedish Ethical Review Authority (Dnr 2022-04968-01) and performed at Capio S:t Görans Hospital in Stockholm. All participants provided oral and written consent. This article is a component of the AICare research project, which seeks to identify and anticipate legal challenges, while also proposing measures to ensure patient safety and enhance public acceptance of AI in healthcare. Conducted as a qualitative, semi-structured interview study, this article will contribute to informing future legal and ethical analyses in this field.

The setting and recruitment

Every day, 350 women, between 40 and 74, undergo their biannual screening examination at the hospital. Participation in the screening program is voluntary and free-of-charge. The participants can reschedule their appointment online or via telephone if necessary. Upon arrival, the participants register their presence using their social security numbers and proceed to the waiting area where they await their turn. Subsequently, radiographers help the women throughout the screening examination, which generates four images, with two views of each breast. They also ask the women questions about clinical symptoms from their breasts, such as new lump(s), secretion, eczema, and skin retraction. The screening examination lasts approximately five minutes. If there are no clinical findings and the mammograms are assessed with no suspicion of cancer, the women receive their mammography results by mail after approximately two weeks.

In the prospective clinical study, ScreenTrustCAD, the results of adding an AI algorithm with Philips equipment to the mammography reading at Capio S:t Göran hospital, was studied. If any reader, AI or radiologists flagged an examination for a suspicious image finding, the examination was discussed at a special meeting called a consensus discussion. On this

For peer review only - http://bmjopen.bmj.com/site/about/guidelines.xhtml

occasion, at least two experienced breast radiologists assessed the mammograms and together decided whether the woman should be declared healthy or recalled for further follow-up. The current interview-based study was conducted at the same time as the ScreenTrustCAD study, i.e., AI was already being used and informed for the women in this study.

Participants (i.e., women participating in mammogram screening) were recruited at the mammography department at Capio S:t Göran Hospital, Sweden. In total, 63 women were invited by the first author (JVJ) to participate in the study before or after the screening procedure. All women were informed in the waiting room and in the consultation room that they were participating in a clinical study. Sixteen women consented, and two of them agreed to attend an interview via Zoom due to a lack of time in conjunction with their screening appointment.

The participants

The participants were very positive about the breast cancer screening program. The majority of the women had never been recalled. Those women who had reported a lump or been recalled expressed that it was very stressful. None of the women had been previously diagnosed and treated for breast cancer. Additional characteristics of the participants are presented in Table 1.

For peer review only - http://bmjopen.bmj.com/site/about/guidelines.xhtml

120 Table 1. The characteristics of the participants (N=16) are presented as frequencies and percentages.

Questions	n (%)
**What is your age?**	
41–50	5 (31.3)
51–60	6 (37.5)
61–70	5 (31.3)
70+	0 (0)
**What is your highest level of education?**	
Less than secondary/high school	1 (6.3)
Secondary/high school	4 (25.0)
University	11 (68.8)
**Have you been called back for a follow-up of your mammogram?**	
Never	13 (81.3)
Once	3 (18.8)
**Compared to other individuals in Sweden of the same age, how do you assess your risk of developing breast cancer in the next 10 years?**	
Slightly lower	1 (6.3)
Neither higher nor lower risk	7 (43.8)
Slightly higher	1 (6.3)
Much higher risk	2 (12.5)
Do not know/have no idea about it	5 (31.3)
**Are you worried about developing breast cancer?**	
Not worried at all	3 (18.8)
A little worried	12 (75.0)
Very worried	1 (6.3)
**Has anyone in your immediate family been affected by breast cancer? (grandmother, aunt, mother, or sister)**	
Yes	2 (12.5)
No	12 (75.0)

For peer review only - http://bmjopen.bmj.com/site/about/guidelines.xhtml

Do not know	2 (12.5)

Data collection

The interviews were performed in February 2023 by the first author (JVJ). They lasted from 15 to 36 minutes each, and they were conducted in Swedish, except one that was conducted in English. We began every interview by asking the participant about her experience of undergoing mammography. We introduced AI in a general context, inquiring about its capabilities. Thereafter, we delineated its application in mammography, illustrating how the AI, a computer program, scans mammogram images and detects irregularities, prompting a response if any deviation from healthy mammograms is detected. Thereafter, we asked about the women's knowledge, thoughts, perceptions and attitudes of the technology in general, especially for evaluating mammograms. In this study, 'perception' refers to participants' individual understanding and viewpoints on AI in breast screening, while 'attitude' encompasses their emotional and evaluative responses towards its use. See Table 2 for the semi-structured interview guide with open-ended questions [19, 20].

For peer review only

Table 2. The interview guide used for the semi-structured interviews primarily focused on open-ended questions to explore the women's perceptions of AI in evaluating their mammograms, although some follow-up questions were more direct to encourage deeper discussion and insight.

**1. Can you start by telling me about your experience undergoing mammography screening?**	
**2. Description of how the mammogram images are assessed and the clinical trial with AI.**	
	What do you think about that? How does it feel? To what extent do you perceive the assessment process as reliable?
	What do you think about AI in the assessment? What emotions does this information evoke?
	Do you feel okay with a doctor being replaced by AI? Could the involvement of AI change your trust in the assessment process? In what way?
	If we imagine that AI would replace both radiologists in the assessment, then you will not receive any other assessment by a doctor. Do you think that is okay (i.e., no human involvement)?
**3. Description of AI and radiologist's work and missing cancer.**	
	What do you think about this? How do you feel about this?
	Would it be different if a doctor or AI misses seeing signs of cancer in the images? If the AI misses it, whose responsibility do you think it is? How do you feel about this?
	It is possible to set AI to signal for everything, even the smallest change, which would make doctors assess many images to determine if a woman should be contacted. As a result, many women will receive a letter stating that they need further tests to rule out cancer, even though not all women will have cancer. What do you think about that? Does it worry you?
	If AI is set to be less sensitive, fewer women will receive such a letter, reducing unnecessary worry. However, this would increase the risk of some women having undetected cancer. On the other hand, healthcare resources would be focused on more severe cases of cancer. What do you think about that? Does it worry you?
	In the long run, imagine AI replacing both radiologists to assess the images. What do you think about that? What risks or concerns do you have?
**4. Description of the development of healthcare through the sharing of healthcare data.**	
	What information about yourself do you consider sensitive? What is acceptable for you to share within healthcare, and what is more sensitive?

For peer review only

	Is a mammogram image (show the image) sensitive if it were to be shared with other hospitals or companies developing these algorithms?
	How do you feel if no other information about you could be linked to your image, only the result of the examination (cancer or not)? Would that be sensitive for you?
**5. Closing questions**	
	After discussing your perspective on using AI in mammography screening, do you have any final thoughts?
	Is there anything else we haven't talked about that you would like to mention?

Analysis

The recorded interviews were transcribed verbatim by a professional transcription company. Subsequently, the audio files were listened to in their entirety to verify the transcriptions. After all the transcripts were read again, meaning units (phrases, sentences, or paragraphs) related to the aim were identified for further scrutiny. The data management and analysis process was facilitated by using Atlas.ti Web [21] Microsoft Excel (2016) as a tool. The next phase of the process entailed comparisons of the meaning units, examining their similarities and differences from the perspective of perception of AI in mammography. Open coding of each meaning unit was added. Two of the interviews were coded simultaneously by both authors (JVJ and ÅG), who jointly discussed what meaning units to identify, interpretations, and formulations of codes; they also created coding frameworks to improve conceptual clarity or transparency in the data analysis. The remaining interviews were coded by JVJ, but regular follow-up meetings were held to discuss new codes. Codes that reflected a similar concept were grouped; sub-categories were formulated, and categories were identified [22, 23] by JVJ and thereafter discussed thoroughly with the other authors (ÅG, FS, and KD); see Table 3. Thematic saturation was attained in alignment with the intended objectives of the data collection [24].

For peer review only

Table 3. An example of the analytical process regarding the perception of using AI as a decision tool evolved from identifying “meaning units” and performing “initial coding” to organizing codes into “sub-categories” and, ultimately, formulating overarching “categories” for a more comprehensive qualitative analysis.

**Meaning unit**	**Initial coding**	**Sub-category**	**Category**
“I think that a human being can... can see, like, ‘Oh, is there something there, then maybe I have to look there, and then I have to look there, and then I have to look there.’ Some kind of reasoning ability.”	Humans can see connections; it’s like detective work	AI is merely a tool	Perceived differences between AI and human assessment
“If it is used as a complement, I think it is good.”	AI should be used as a complement	Human in the loop	Requirements when using AI in mammography
“...anonymization is important, ensuring that the AI company or any other party does not have access to personal data or information about individuals. It’s crucial to protect privacy and prevent sensitive data from being linked to specific individuals.”	Consider sharing deidentified mammography images with AI companies	Trust in data sharing varies in routine care, research, and industry	Trust when implementing AI in mammography

Patient and public involvement

None.

For peer review only

Results

The results below describe the perceptions and attitudes expressed by the participants regarding AI in healthcare. We identified 318 meaning units in total. When analyzing them, seven sub-categories emerged, which were categorized into three main categories: perceived differences between AI and human assessment, trust when implementing AI in mammography, and requirements when using AI in mammography. An overview of these sub-categories and main categories is presented in Table 3. These will be described and illustrated using quotes in the following sections.

Table 3. The categories and sub-categories regarding participants' perceptions of taking part in triage for mammography using AI.

**Category**	**Sub-category**
Perceived differences between AI and human assessment	AI is merely a tool
	The difference in error tolerance
Attitudes when implementing AI in mammography	Trust in the healthcare system gives confidence in AI
	Willingness to data sharing varies in routine care, research, and industry
Requirements when using AI in mammography	Evaluation of AI
	Human in the loop
	The trade-off between being worried about possible cancer and detecting cancer

Category: Perceived differences between AI and human assessment

Within this category, the participants talked about the positive and negative characteristics of AI and human assessment. They also talked about how their moral expectations differed between AI and humans regarding error tolerance.

For peer review only

Sub-category: AI is merely a tool

The participants were familiar with AI to varying degrees, primarily through their interactions with search engines and recommendation systems on the internet. In addition to the positive aspects, they had heard stories about AI being used to cheat when writing papers and exams. Some participants expressed excitement about the development of AI, recognizing its potential benefits. However, one participant cautioned against the excessive hype, stating that AI should not be equated with the futuristic concept portrayed by some. Despite the generally positive sentiment toward the utilization of AI in mammography, a subset of participants voiced reservations regarding their limited understanding of the underlying mechanisms of AI. They expressed a sense of ambivalence toward forming a definitive opinion on the benefits or drawbacks of AI implementation in mammography. After a brief presentation on how AI works at the clinic, they were all capable of expressing their attitudes toward AI. They made clear distinctions between the characteristics and abilities of an AI compared with a human; they often drew upon what humans are capable of when describing what AI can or cannot do.

> *No, but I mean, can you build a machine that is that skilled? That's what I'm thinking. In addition, the human eye and experience of how things can look; I don't think you can just cram all of that into a robot, you know.* (Participant 7)

The women acknowledged and appreciated that AI has the potential to identify patterns that may elude the human eye and respond to deviations from the norm. They perceived that AI could be a powerful tool, helping radiologists complement their work. Some expected that the use of AI would save resources for the healthcare system and give radiologists the possibility of performing other tasks. Some perceived that the AI to a great extent would complement the radiologists. They believed that AI is self-learning, quick, and will never get tired. Some

For peer review only

For peer review only - http://bmjopen.bmj.com/site/about/guidelines.xhtml

participants held the belief that AI technology will continue to evolve over time, surpassing its current capabilities and becoming even more advanced. They expressed that the utilization of AI represents the future of healthcare. Consequently, humans and AI were perceived as complementary entities, working together synergistically.

Nevertheless, a prevailing skepticism regarding the current capabilities of AI was evident among many participants. One expressed a general cautious perspective toward technology, acknowledging its benefits as long as it functions properly. However, some raised concerns about the sustainability of AI effectiveness in the long run. Some participants expressed uncertainty regarding AI's ability to detect all cancers, stating that AI might have difficulty with certain more uncommon conditions, such as breast implants or dense breast tissue. They also doubted whether AI could be good enough to replace the two radiologists currently assessing the mammography images. On the other hand, AI could be good at detecting things that the human eye cannot. However, participants expressed uncertainty regarding the specific areas in which AI excels beyond the capabilities of the human brain.

> *I also think that a human has the ability to... some form of consequential thinking. I mean, a reasoning ability that AI possibly doesn't have.* (Participant 6)

Some of the major strengths attributed to humans included their holistic perspective, considering various factors related to the individual woman, and making connections between different pieces of information. Humans were also perceived as capable of thinking about consequences, conducting investigative work, and demonstrating greater imagination. Overall, human abilities were perceived in a more positive sense compared to AI. Many participants, however, expressed a willingness to accept the use of AI if it could outperform radiologists in terms of detecting more cases of cancer. The primary concern for them was the timely and accurate identification of cancer, regardless of the method used. However, they

For peer review only - http://bmjopen.bmj.com/site/about/guidelines.xhtml

For peer review only

also strongly believed that human involvement adds an irreplaceable element that AI currently lacks and perhaps never will possess. Participants emphasized that humans bring unique qualities and abilities to the screening process that go beyond mere detection, such as intuition, empathy, and contextual understanding. While they acknowledged the potential of AI, they maintained that human touch and contribution are invaluable and offer something distinct that AI cannot yet replicate.

> *I'm not a hundred percent convinced that the human eye can be replaced because there are... we also had scanning microscopes and things like that. That was a hundred years ago now... but, I mean, it [AI] is an aid.* (Participant 3)

Sub-category: The difference in error tolerance

The ability of AI to detect cancer in mammography was consistently emphasized by all participants, and the failure to detect cancer was perceived as devastating. However, their acceptance of failure to detect cancer differed between radiologists and AIs, having a much higher acceptance for mistakes made by radiologists than by an AI. This was because participants felt that to err is human – and therefore understandable. In contrast, the majority expected that technology, including AI, should not make mistakes when used in healthcare. They believed that AI, to be acceptable upon implementation, should function well, maintain a high level of security, and operate without errors. They questioned the rationale behind implementing AI if it did not meet these criteria. Two participants specifically highlighted the potential consequences of AI-making mistakes that could be catastrophic due to the systematic nature of errors. In contrast, they pointed out that if a single radiologist were to miss a few cases of cancer in a day, the impact would be comparatively limited. The participants expressed a range of negative emotions, including feeling bad, disappointment, and cheating if AI was to make a mistake. One participant held a pessimistic view of

For peer review only - http://bmjopen.bmj.com/site/about/guidelines.xhtml

technology and questioned whether it could guarantee reliable performance at all times, despite acknowledging its current functionality.

> *Yes, because we all know that a technology […] it should be a hundred percent...* (Participant 2)

A few participants expressed a more nuanced perspective, acknowledging that neither humans nor AI can be expected to be 100% accurate all the time. They recognized that AI relies on data and statistical predictions, which inherently introduces the possibility of errors. These participants emphasized that no system, whether human or AI, is infallible, and both can potentially make mistakes in their assessments.

The participants held high expectations of the radiologists and regarded them as having ultimate responsibility for the final results. Additionally, some participants believed that if AI were to make a mistake, it would be the responsibility of humans, as AI is viewed as a tool incapable of being held accountable for its decisions.

> *[…] It is always the medically responsible physician, the radiologist, who is accountable. We can't blame the technology. We must have technology that is tested and that we believe in and trust. It's always the medically responsible physician, in my opinion. Where else should the responsibility lie, I wonder. That's how I think.* (Participant 4)

However, one respondent expressed an opposing viewpoint, suggesting that if a radiologist were to make a mistake, it could be attributed to a flaw in the device or image-computer system. Another participant highlighted the importance of the radiologist not disregarding the indications provided by AI when it signals a case of potential cancer. Overall, the participants' perceptions varied regarding the issue of responsibility.

For peer review only - http://bmjopen.bmj.com/site/about/guidelines.xhtml

Category: Attitudes when implementing AI in mammography

This category and its sub-categories concerned women's attitudes in sharing mammography images with different actors, as well as trust in the healthcare system's implementation of AI. Here, we identified two sub-categories.

Sub-category: Trust in the healthcare system gives confidence in AI

This sub-category predominantly reflects the participants' strong trust in the healthcare system in regard to implementing AI in routine practices. All the participants expressed a high level of confidence that healthcare providers would take the necessary steps to deliver the best possible care, even if it involved the implementation of AI.

> *I trust that hospitals do it to the best of their ability.* (Participant 6)

While some participants acknowledged the potential for unwanted motivations, such as cost-saving measures behind AI implementation, they still expressed trust that both healthcare providers and developers of AI tools have the intention of saving lives and improving patient outcomes. Some participants even indicated that if healthcare professionals have good intentions in utilizing AI, they do not feel the need to have detailed information regarding its use.

> *No, I think experts must have examined and evaluated that, so no, I would probably trust... I mean, I think if they have chosen it, it is because it's very effective and accurate.* (Participant 9)

Sub-category: Willingness to data sharing varies in routine care, research, and industry

The participants consistently expressed their willingness to share their health information within the healthcare system setting. Furthermore, many participants held a strong belief that different healthcare units should collaborate and share information more extensively than they currently do, emphasizing that such sharing is crucial to ensure patient safety. However,

For peer review only - http://bmjopen.bmj.com/site/about/guidelines.xhtml

in regard to sharing health-related data or personal data with companies, the participants were more reluctant. For some, it was perceived as a great violation of their privacy. The participants felt that mammogram images of their breasts were less controversial if they were completely anonymized and not connected to them as individuals. Anonymization of the mammograms helped to alleviate concerns regarding privacy and personal identification.

> *No, I wouldn't have any issues with that [sharing mammograms] as long as it can't be linked to me as a specific individual.* (Participant 6)

For peer review only

However, for many of the participants, providing consent to share the data were viewed as necessary and connected to their ability to influence that the mammograms would be used for a meaningful purpose.

> *On the other hand, if it's a research project or a pilot project, then you want to know that you're participating in such a project. However, if it's part of the established methodology at hospitals... I don't interfere with that.* (Participant 6)

> *I would like to know beforehand so that they don't just send it off without informing me. It doesn't feel entirely right. I would expect to be informed about it, though.* (Participant 8)

They were willing to consent if the purpose was to save lives, develop new medical devices, or conduct research. Conversely, if the data usage was intended for commercial companies or advertising purposes, they expressed a strong reluctance to share their data.

Category: Requirements when using AI in mammography

This category reflects the prerequisites expressed by our participants regarding the use of AI in the mammography screening process.

For peer review only - http://bmjopen.bmj.com/site/about/guidelines.xhtml

Sub-category: Evaluation of AI

This sub-category related to the paramount evaluation of AI in multiple aspects. Participants highlighted the need to evaluate AI's cancer detection performance compared to radiologists and the overall integration process and integration process. Participants stressed evaluating professionals' perspectives, AI image selection criteria, and the impact on radiologists' skills. They underscored the importance of radiologists' continuous training, decision-makers' understanding of AI's implications, and the need for control and monitoring to ensure AI's accuracy and relevance to the Swedish context.

The participants expressed concern that decision-makers might focus solely on certain aspects, such as cost savings, when evaluating AI, potentially overlooking other important factors, such as providing comprehensive training for specialists.

The participants agreed that the evaluation of the AI's performance itself needs to focus on the ability of AI to keep the margin of error down and evaluate to what extent it performs better.

> *I think it's possible to evaluate the error margins and compare the percentage of correct or incorrect findings between the two. Therefore, I believe that would be beneficial.* (Participant 5)

Sub-category: Human in the loop

All participants preferred humans over AI; however, they expressed that combining AI and humans would be a complement to each other. The participants emphasized the need for human oversight due to their perception of the unique qualities possessed by radiologists and humans; even though AI is a powerful tool, it requires human control. They expressed the desire to harness the combined strengths of both humans and AI. Moreover, participants believed that a collaborative approach, where AI assists radiologists in detecting abnormalities, would yield the best outcomes.

For peer review only - http://bmjopen.bmj.com/site/about/guidelines.xhtml

No, but I think... as long as there is a human factor involved, I believe it can be very effective. (Participant 6)

Some of the participants identified the risk that using AI too much would lead to a decline in learning skill development for the radiologist. They reasoned that if AI gives too many false positives, it will create a misleading foundation for radiologists to identify cancer cases. They expressed concerns that if AI solely performed the initial assessment, radiologists would only discuss a selected sample of images identified by AI. One respondent had a different perspective and was less concerned about radiologists losing competence. Instead, she understood that the development of AI requires radiologists to continuously improve their skills. She recognized that to effectively contribute to the development and training of AI systems, radiologists must stay updated and enhance their expertise. This viewpoint highlights the symbiotic relationship between radiologists and AI, where ongoing skill development and collaboration contribute to the advancement of both fields.

But it is still the case that radiologists constantly need to enhance their competence to develop AI. Because it's the people who collect the data; it's not like an external force that does it. (Participant 4)

Some expressed a hopeful perspective regarding the combination of AI and human involvement in preventing potential errors. They perceived that there is too much uncertainty regarding the performance of AI, which led them to believe that relying solely on AI at the present moment would be a substantial leap. They expressed fears that something will be lost and that the radiologists will undermine their role in the diagnostic process; AI can serve as a support tool, but a human needs to be in control. All participants thought it was acceptable to implement AI as long as one radiologist was still involved in the first round. However, one participant expressed that it was compulsory to like digitization, as it is a reflection of our society's direction. She expressed a preference for a collaborative approach, desiring the

For peer review only - http://bmjopen.bmj.com/site/about/guidelines.xhtml

presence of a radiologist in the process and appreciation for human contact and interaction in the process.

Sub-category: The trade-off between being worried about possible cancer and detecting cancer

The participants strongly expressed that failing to detect a case of cancer is terrible, and they emphasized the importance of healthcare providers doing everything possible to ensure early detection. They recognized the trade-off between causing increased worry among women due to more false positive cases and the potential risk of missing a cancer diagnosis after explaining that this was the reality. However, they expressed a clear preference for prioritizing the detection of potential cancer cases over minimizing false positives. They believed that it was better for more individuals to experience the anxiety of false-positive results than for a potential case of cancer to go undetected.

> *I think we should be a bit more... we should be able to live a bit more in reality [not being afraid of bad news]. And if we can get the help [hinder cancer] that it entails, that I have to go for an extra appointment, then I would just be thankful and accept it. I'm not inclined in the other direction. No, I would rather go for the extra appointment than not do it.* (Participant 4)

Participants agreed on setting AI to a highly sensitive level for better cancer detection and emphasized the need for transparent communication about AI use in Swedish healthcare. They believed women would accept AI if it improved cancer detection and advocated for detailed information in referral letters about potential increased callbacks due to AI's sensitivity.

For peer review only

> Then, they must communicate that 'the experience has shown that about thirty percent of those who have this develop cancer, or zero or twenty or forty.' Something like that. I think it's good to communicate that, if you ask me. *(Participant 1)*

Participants preferred immediate follow-ups to lessen anxiety, viewing screening as an act of solidarity and accepting false positives for timely cancer treatment for others. They saw the initial resource investment in AI as ultimately cost-saving through early detection. Emphasizing transparency to maintain trust, they indicated they wouldn't opt out of mammography with AI integration, underscoring the importance of open communication in healthcare AI.

For peer review only

Discussion

The main findings

The main findings of this study demonstrate that participants perceive AI as a valuable tool in mammography, recognizing the potential benefits and its ability to complement the work performed by radiologists. However, there are concerns about participants' limited understanding of AI and reservations about its current capabilities. Participants stressed human involvement, transparent screening, and evaluating AI's ability to detect cancer. They prioritized finding potential cancers over reducing false positives and believed that women would accept AI if it detected more cases. The emphasis on cancer detection, while crucial, poses challenges due to limited healthcare resources. Lowering sensitivity thresholds raises ethical questions about costs and personnel. It is imperative to explore the lessons learned from Sweden's mammography program and consider the ethical, defensible trade-offs, especially in resource allocation. Striking a balance between optimal detection and efficient resource use remains a pivotal area for healthcare policy discussions.

The results underscore the importance of a collaborative approach, where AI and radiologists complement each other and where radiologists maintain a vital role in the diagnostic process by contributing with their "human capabilities." These findings align with other patient groups, where participants emphasized the need for, and value of, information, even if it is not used in medical decision-making [25]. The study shows that it is crucial to inform patients about the use of AI as a straightforward clinical implication, aiming to maintain participants' trust. Additionally, the study highlights that human involvement in medical care is highly valued, and it would be difficult to replace it with AI. This is a consistent theme identified in studies outside the realm of mammography [26] [27].

For peer review only

Discussion of each finding

Within the first category in our study, we discovered perceptions regarding differences between AI and human assessment. Participants were cautiously optimistic about AI in healthcare, acknowledging its benefits but expressing reservations due to current limitations in detecting all cancers and conditions. Interestingly, these views align with those of participants in prior studies where concerns about AI replacing radiologists entirely, apprehensions regarding the absence of a 'human touch' in the diagnostic process [14, 17], and accountability issues hindered the acceptance of AI-driven medicine [28, 29]. Moreover, our study emphasizes the importance of retaining human expertise and clear lines of responsibility in breast cancer screening. Participants underlined these aspects, emphasizing the need for a specific framework, although such guidelines are still under development. The National Board of Health and Welfare in Sweden has initiated work on guidelines for AI in healthcare,[30] a step in the right direction. This emphasis aligns with prior research stressing the importance of preserving human involvement in breast screening [31, 32]. It highlights the complexity of AI perception in healthcare, emphasizing the necessity for tailored guidelines that address regulatory and ethical concerns while catering to the specific demands of distinct medical fields.

It is particularly intriguing to note that participants in our study placed significant emphasis on the consequences of technological errors. Most women held high expectations for AI, anticipating its flawless performance, unwavering security, and a complete absence of errors. Strikingly, when contemplating the prospect of AI making mistakes, participants expressed strong negative emotions. This contrasts with the perceptions of healthcare professionals, where a certain degree of error was deemed more acceptable, attributed to the inherent human factor [33, 34]. People in general tolerate fewer mistakes made by technical products than mistakes made by humans. A pivotal observation was the varying perspectives on responsibility among the participants. While many ascribed the ultimate responsibility for

For peer review only - http://bmjopen.bmj.com/site/about/guidelines.xhtml

potential errors to radiologists, a subset viewed AI as an entity lacking in accountability. These attitudes describe the intricate interplay between human trust, the technology's reliability, and the attribution of responsibility in the context of AI implementation within healthcare. This divergence in tolerance towards errors by humans versus AI underscores a psychological aspect of trust in healthcare. Participants view human errors as part of learning, but see AI mistakes as systemic flaws, challenging trust in technology. This dichotomy raises critical questions about how we educate and prepare the public for the realistic capabilities and limitations of AI in healthcare, balancing technological optimism with a pragmatic understanding of AI as an evolving tool rather than an infallible solution.

In our study, the category "Attitudes when Implementing AI in Mammography" revealed that participants generally exhibited trust in the integration of AI into routine mammography practices. They believed that healthcare providers and AI developers shared a common goal of saving lives and enhancing patient outcomes. However, willingness had boundaries, as participants were reluctant to share health information with external companies, considering it an intrusion into their private lives.

The participants expressed that maintaining the confidentiality of the mammogram images played a pivotal role in addressing privacy concerns, making participants more willing to share their data as long as it could not be traced back to them as individuals. Consent for data sharing was often contingent on meaningful purposes, such as life-saving measures or research initiatives. Conversely, participants expressed hesitation about sharing data for commercial or advertising purposes. This aligns with previous studies on people's attitudes toward data sharing [35]. There is a risk that the participants do not comprehend the necessity of sharing data with private entities to advance technology.

In summary, our study underscores the pivotal role of attitudes in AI implementation in healthcare. Trust, rooted in transparent collaboration between healthcare providers and participants, is fundamental. Recognizing its importance is crucial in the complex realm of AI

integration. Collaborative efforts, informed by human rights frameworks and practical recommendations, are essential. These actions not only preserve but also nurture trust, ensuring the inclusive and effective application of AI technology in healthcare [36, 37].

In the third category, participants expressed their thoughts on prerequisites for the implementation of AI in mammography screening. In line with these expressed prerequisites, it is evident that the implementation of AI in healthcare, particularly in mammography screening, has brought to the forefront a multitude of ethical considerations. These considerations closely align with the broader discussions in the literature surrounding the ethical dimensions of AI integration [11, 38]. These include evaluating AI's performance compared with that of radiologists, ensuring transparency in the screening process, and addressing concerns about the potential loss of expertise among radiologists. The participants stressed the importance of ongoing training for radiologists and urged decision-makers to consider factors beyond cost savings. It is therefore important that there is a solution for unexperienced radiologists to undergo training in mammography screening. Trust in AI is expected to increase as familiarity with its capabilities grows. This was also observed among the radiologists when the ScreenTrust CAD study started, as recall rates increased during the first few months and later normalized. Although participants in that study preferred human involvement in the screening process, they recognized the potential of AI in detecting abnormalities and believed that a combination of both would yield the best outcomes, which the ScreenTrust CAD study confirmed [9]. Participants prioritized the early detection of cancer, even if it meant accepting more false-positive results and having to return for further follow-up examinations. Participants saw the implementation of AI as initially requiring additional resources but ultimately saving money by detecting cancers earlier with fewer resources.

Strengths and limitations

This study, focusing on women's perceptions and attitudes toward AI in mammography, offers insights for shaping ethical frameworks and legal guidelines in AI healthcare applications. While not a bioethical analysis per se, it provides valuable input for future ethical and legal considerations in AI integration in healthcare. However, the findings may only be applicable to this AI tool at Capio S:t Görans Hospital with Philips equipment. The study excluded other AI-developed tools, which may limit the transferability of the results. One major strength of our study is that we interviewed women who were actively undergoing mammography, where AI was utilized as a third reviewer. This means that we have addressed the hypothetical bias commonly encountered in studies [39].

There are potential limitations in self-reporting bias and subjectivity in interview-based studies. To address these limitations, the researchers encouraged honest and candid responses, maintained confidentiality, reflected on their own perspectives and biases, sought input from colleagues, and used coding frameworks to improve objectivity in data analysis [40]. Considering transferability, we identify a limitation: our study predominantly intercepted participants who exhibited high trust in healthcare, a sentiment likely to influence their confidence in the implementation of AI. To address this gap, we recommend examining specific demographic groups to gain comprehensive insights. Some people with low trust in conventional healthcare might also be skeptical about AI, while others might perceive AI as more objective, leading to higher trust. This viewpoint is supported by Yang et al.,[26] indicating varied levels of trust based on demographics. Understanding these dynamics is vital to grasp societal attitudes toward AI in healthcare.

For peer review only

Relevance

In summary, this study's findings hold significant relevance for the ongoing debate surrounding AI in healthcare, especially within breast cancer screening programs. This highlights the essential role of trust in AI and the necessity of retaining human involvement in making decisions regarding mammogram images. These insights are vital for ensuring the effective and well-received implementation of AI, emphasizing transparent communication and comprehensive patient education regarding the role of AI in breast cancer screening. In this study, participants clearly emphasized the ethical principles of fairness, privacy, responsibility, and accuracy while underscoring the competence of radiologists and the importance of transparency. Their overall positive attitude toward the technology is evident.

Conclusion

The findings of this study indicate that women who participate in mammography view AI as one of many tools in the healthcare system, not as a standalone solution. They acknowledge its potential but emphasize its role in complementing existing practices rather than replacing the radiologists entirely. However, if healthcare professionals determine that AI functions equally or even better than traditional screening processes, then the participants find the implementation of AI without explicit consent acceptable. Notably, women prefer AI to be sensitive in detecting potential cancer, even if it may lead to an increased level of unnecessary worry and fear. Nevertheless, effective communication regarding the role and limitations of AI is crucial to help patients understand its purpose and potential outcomes in healthcare and to maintain their trust.

For peer review only - http://bmjopen.bmj.com/site/about/guidelines.xhtml

References

1. Törnberg, S., et al., *A pooled analysis of interval cancer rates in six European countries.* European Journal of Cancer Prevention, 2010. **19**(2): p. 87-93.
2. Dembrower, K., et al., *Effect of artificial intelligence-based triaging of breast cancer screening mammograms on cancer detection and radiologist workload: a retrospective simulation study.* The Lancet Digital Health, 2020. **2**(9): p. e468-e474.
3. Buch, V.H., I. Ahmed, and M. Maruthappu, *Artificial intelligence in medicine: current trends and future possibilities.* 2018. **68**(668): p. 143-144.
4. Horgan, D., et al., *Artificial Intelligence: Power for Civilisation – and for Better Healthcare.* Public Health Genomics, 2019. **22**(5-6): p. 145-161.
5. Chan, H.-P., R.K. Samala, and L.M. Hadjiiski, *CAD and AI for breast cancer—recent development and challenges.* 2020. **93**(1108): p. 20190580.
6. Schaffter, T., et al., *Evaluation of Combined Artificial Intelligence and Radiologist Assessment to Interpret Screening Mammograms.* JAMA Network Open, 2020. **3**(3): p. e200265-e200265.
7. McKinney, S.M., et al., *International evaluation of an AI system for breast cancer screening.* Nature, 2020. **577**(7788): p. 89-94.
8. Yoon, J.H., et al., *Standalone AI for Breast Cancer Detection at Screening Digital Mammography and Digital Breast Tomosynthesis: A Systematic Review and Meta-Analysis.* Radiology, 2023. **307**(5): p. e222639.
9. Dembrower, K., et al., *Artificial intelligence for breast cancer detection in screening mammography in Sweden: a prospective, population-based, paired-reader, non-inferiority study.* J The Lancet Digital Health, 2023.
10. Lång, K., et al., *Artificial intelligence-supported screen reading versus standard double reading in the Mammography Screening with Artificial Intelligence trial (MASAI): a clinical safety analysis of a randomised, controlled, non-inferiority, single-blinded, screening accuracy study.* J The Lancet Oncology, 2023. **24**(8): p. 936-944.
11. Carter, S.M., et al., *The ethical, legal and social implications of using artificial intelligence systems in breast cancer care.* The Breast, 2020. **49**: p. 25-32.
12. Omoumi, P., et al., *To buy or not to buy—evaluating commercial AI solutions in radiology (the ECLAIR guidelines).* European Radiology, 2021. **31**(6): p. 3786-3796.
13. McCradden, M.D., T. Sarker, and P.A. Paprica, *Conditionally positive: a qualitative study of public perceptions about using health data for artificial intelligence research.* 2020. **10**(10): p. e039798.
14. Lennox-Chhugani, N., et al., *Women's attitudes to the use of AI image readers: a case study from a national breast screening programme.* BMJ health & care informatics, 2021. **28**(1): p. e100293.
15. Ongena, Y.P., et al., *Patients' views on the implementation of artificial intelligence in radiology: development and validation of a standardized questionnaire.* European Radiology, 2020. **30**(2): p. 1033-1040.
16. Haan, M., et al., *A Qualitative Study to Understand Patient Perspective on the Use of Artificial Intelligence in Radiology.* Journal of the American College of Radiology, 2019. **16**(10): p. 1416-1419.
17. Ongena, Y.P., et al., *Artificial Intelligence in Screening Mammography: A Population Survey of Women's Preferences.* Journal of the American College of Radiology, 2021. **18**(1, Part A): p. 79-86.
18. Ploug, T. and S. Holm, *The right to refuse diagnostics and treatment planning by artificial intelligence.* Medicine, Health Care and Philosophy, 2020. **23**(1): p. 107-114.

For peer review only - http://bmjopen.bmj.com/site/about/guidelines.xhtml

19. Kvale, S. and S. Brinkmann, *Interviews: Learning the craft of qualitative research interviewing*. 2009: Sage.
20. Krueger, R.A. and M.A. Casey, *Focus groups: A practical guide for applied research*. 2014: Sage Publications.
21. ATLAS.ti Scientific Software Development GmbH. *ATLAS.ti Web - The easiest way to uncover qualitative insights*. 2022 [cited 2022; Available from: https://atlasti.com/atlas-ti-web.
22. Burnard, P., *Writing a qualitative research report.* Nurse Education Today, 2004. **24**(3): p. 174-179.
23. Burnard, P., et al., *Analysing and presenting qualitative data.* British Dental Journal, 2008. **204**(8): p. 429-432.
24. Malterud, K., V.D. Siersma, and A.D. Guassora, *Sample size in qualitative interview studies: guided by information power.* J Qualitative health research, 2016. **26**(13): p. 1753-1760.
25. Grauman, Å., et al., *Personalizing precision medicine: Patients with AML perceptions about treatment decisions.* Patient Education and Counseling, 2023. **115**: p. 107883.
26. Yang, K., et al., *Attitudes Of Chinese Cancer Patients Toward The Clinical Use Of Artificial Intelligence.* Patient Prefer Adherence, 2019. **13**: p. 1867-1875.
27. Juravle, G., et al., *Trust in artificial intelligence for medical diagnoses.* Prog Brain Res, 2020. **253**: p. 263-282.
28. Tamori, H., et al., *Acceptance of the use of artificial intelligence in medicine among Japan's doctors and the public: a questionnaire survey.* J JMIR human factors, 2022. **9**(1): p. e24680.
29. Pesapane, F., et al., *Women's perceptions and attitudes to the use of AI in breast cancer screening: A survey in a cancer referral centre.* J The British Journal of Radiology, 2023. **95**(1141): p. 20220569.
30. Österberg, M. and L. Lindsköld. *AI for better health*. 2018; Available from: https://swelife.se/wp-content/uploads/2021/01/AI_for_better_health_final.pdf.
31. Carter, S.M., et al., *Australian women's judgements about using artificial intelligence to read mammograms in breast cancer screening.* 2023. **9**: p. 20552076231191057.
32. Jonmarker, O., et al., *The future of breast cancer screening: what do participants in a breast cancer screening program think about automation using artificial intelligence?* Acta Radiologica Open, 2019. **8**(12): p. 2058460119880315.
33. Hendrix, N., et al., *Artificial intelligence in breast cancer screening: primary care provider preferences.* Journal of the American Medical Informatics Association, 2020. **28**(6): p. 1117-1124.
34. Zhang, Z., et al., *Patients' perceptions of using artificial intelligence (AI)-based technology to comprehend radiology imaging data.* J Health Informatics Journal, 2021. **27**(2): p. 14604582211011215.
35. Viberg Johansson, J., et al., *Publics' preferences for sharing health data: a discrete choice experiment.* JMIR Medical Informatics 2021. **in press**.
36. Gibbons, E.D. and S. Magazine, *Toward a more equal world: the human rights approach to extending the benefits of artificial intelligence.* J IEEE Technology, 2021. **40**(1): p. 25-30.
37. Secinaro, S., et al., *The role of artificial intelligence in healthcare: a structured literature review.* J BMC medical informatics, 2021. **21**: p. 1-23.
38. Murphy, K., et al., *Artificial intelligence for good health: a scoping review of the ethics literature.* BMC Medical Ethics, 2021. **22**(1): p. 14.
39. Loomis, J.J.J.o.E.S., *What's to know about hypothetical bias in stated preference valuation studies?* 2011. **25**(2): p. 363-370.
40. Lincoln, Y.S. and E.G. Guba, *Naturalistic inquiry*. 1984, Beverly Hills, CA: SAGE.

For peer review only - http://bmjopen.bmj.com/site/about/guidelines.xhtml

667

For peer review only - http://bmjopen.bmj.com/site/about/guidelines.xhtml

COREQ (COnsolidated criteria for REporting Qualitative research) Checklist

A checklist of items that should be included in reports of qualitative research. You must report the page number in your manuscript where you consider each of the items listed in this checklist. If you have not included this information, either revise your manuscript accordingly before submitting or note N/A.

Topic	Item No.	Guide Questions/Description	Reported on Page No.
**Domain 1: Research team and reflexivity**			
Personal characteristics			
Interviewer/facilitator	1	Which author/s conducted the interview or focus group?	7,29
Credentials	2	What were the researcher's credentials? E.g. PhD, MD	1
Occupation	3	What was their occupation at the time of the study?	1
Gender	4	Was the researcher male or female?	1
Experience and training	5	What experience or training did the researcher have?	1
Relationship with participants			
Relationship established	6	Was a relationship established prior to study commencement?	4
Participant knowledge of the interviewer	7	What did the participants know about the researcher? e.g. personal goals, reasons for doing the research	4, 5
Interviewer characteristics	8	What characteristics were reported about the inter viewer/facilitator? e.g. Bias, assumptions, reasons and interests in the research topic	6
**Domain 2: Study design**			
Theoretical framework			
Methodological orientation and Theory	9	What methodological orientation was stated to underpin the study? e.g. grounded theory, discourse analysis, ethnography, phenomenology, content analysis	7
Participant selection			
Sampling	10	How were participants selected? e.g. purposive, convenience, consecutive, snowball	4, 5
Method of approach	11	How were participants approached? e.g. face-to-face, telephone, mail, email	5
Sample size	12	How many participants were in the study?	4
Non-participation	13	How many people refused to participate or dropped out? Reasons?	4
Setting			
Setting of data collection	14	Where was the data collected? e.g. home, clinic, workplace	4
Presence of non-participants	15	Was anyone else present besides the participants and researchers?	7
Description of sample	16	What are the important characteristics of the sample? e.g. demographic data, date	6
Data collection			
Interview guide	17	Were questions, prompts, guides provided by the authors? Was it pilot tested?	8
Repeat interviews	18	Were repeat inter views carried out? If yes, how many?	no
Audio/visual recording	19	Did the research use audio or visual recording to collect the data?	9
Field notes	20	Were field notes made during and/or after the inter view or focus group?	yes
Duration	21	What was the duration of the inter views or focus group?	7
Data saturation	22	Was data saturation discussed?	9
Transcripts returned	23	Were transcripts returned to participants for comment and/or	no

For peer review only

Topic	Item No.	Guide Questions/Description	Reported on Page No.
		correction?	
**Domain 3: analysis and findings**			
Data analysis			
Number of data coders	24	How many data coders coded the data?	11
Description of the coding tree	25	Did authors provide a description of the coding tree?	10, 11
Derivation of themes	26	Were themes identified in advance or derived from the data?	9
Software	27	What software, if applicable, was used to manage the data?	9
Participant checking	28	Did participants provide feedback on the findings?	no
Reporting			
Quotations presented	29	Were participant quotations presented to illustrate the themes/findings? Was each quotation identified? e.g. participant number	11
Data and findings consistent	30	Was there consistency between the data presented and the findings?	11
Clarity of major themes	31	Were major themes clearly presented in the findings?	11
Clarity of minor themes	32	Is there a description of diverse cases or discussion of minor themes?	23

Developed from: Tong A, Sainsbury P, Craig J. Consolidated criteria for reporting qualitative research (COREQ): a 32-item checklist for interviews and focus groups. *International Journal for Quality in Health Care*. 2007. Volume 19, Number 6: pp. 349 – 357

Once you have completed this checklist, please save a copy and upload it as part of your submission. DO NOT include this checklist as part of the main manuscript document. It must be uploaded as a separate file.

For peer review only

Women's perceptions and attitudes toward the use of AI in mammography in Sweden: a qualitative interview study

Journal:	*BMJ Open*
Manuscript ID	bmjopen-2024-084014.R1
Article Type:	Original research
Date Submitted by the Author:	31-Jan-2024
Complete List of Authors:	Viberg Johansson, Jennifer; Uppsala University Department of Public Health and Caring Sciences, Centre for Research Ethics & Bioethics (CRB)<br>Dembrower, Karin; Capio Saint Gorans Hospital<br>Strand, F; Karolinska Institute, Department of Oncology-Pathology<br>Grauman, Å; Uppsala Universitet, Department of Public Health and Caring Sciences
**Primary Subject Heading**:	Radiology and imaging
Secondary Subject Heading:	Ethics
Keywords:	RADIOLOGY & IMAGING, Patient Participation, Patient Satisfaction

SCHOLARONE™
Manuscripts

I, the Submitting Author has the right to grant and does grant on behalf of all authors of the Work (as defined in the below author licence), an exclusive licence and/or a non-exclusive licence for contributions from authors who are: i) UK Crown employees; ii) where BMJ has agreed a CC-BY licence shall apply, and/or iii) in accordance with the terms applicable for US Federal Government officers or employees acting as part of their official duties; on a worldwide, perpetual, irrevocable, royalty-free basis to BMJ Publishing Group Ltd ("BMJ") its licensees and where the relevant Journal is co-owned by BMJ to the co-owners of the Journal, to publish the Work in this journal and any other BMJ products and to exploit all rights, as set out in our licence.

The Submitting Author accepts and understands that any supply made under these terms is made by BMJ to the Submitting Author unless you are acting as an employee on behalf of your employer or a postgraduate student of an affiliated institution which is paying any applicable article publishing charge ("APC") for Open Access articles. Where the Submitting Author wishes to make the Work available on an Open Access basis (and intends to pay the relevant APC), the terms of reuse of such Open Access shall be governed by a Creative Commons licence – details of these licences and which Creative Commons licence will apply to this Work are set out in our licence referred to above.

Other than as permitted in any relevant BMJ Author's Self Archiving Policies, I confirm this Work has not been accepted for publication elsewhere, is not being considered for publication elsewhere and does not duplicate material already published. I confirm all authors consent to publication of this Work and authorise the granting of this licence.

For peer review only

Journals: BMJ Open

Title: Women's perceptions and attitudes toward the use of AI in mammography in Sweden: a qualitative interview study

Abstract

Background: Understanding women's perspectives can help to create an effective and acceptable AI implementation for triaging mammograms, ensuring a high proportion of screening-detected cancer. This study aimed to explore Swedish women's perceptions and attitudes toward the use of AI in mammography.

Method: Semi-structured interviews were conducted with sixteen women (N=16) recruited in the spring of 2023 at Capio S:t Görans Hospital, Sweden, during an ongoing clinical trial of AI in screening (ScreenTrustCAD, NCT 04778670) with Philips equipment. The interview transcripts were analyzed using inductive thematic content analysis.

Results: In general, women viewed AI as an excellent complementary tool to help radiologists in their decision-making, rather than a complete replacement of their expertise. To trust the AI, the women requested a thorough evaluation, transparency about AI usage in healthcare, and the involvement of a radiologist in the assessment. They would rather be more worried because of being called in more often for scans than risk having overlooked a sign of cancer. They expressed substantial trust in the healthcare system if the implementation of AI was to become a standard practice.

Conclusion: The findings suggest that the interviewed women, in general, hold a positive attitude toward the implementation of AI in mammography; nonetheless, they expect and demand more from an AI than a radiologist. Effective communication regarding the role and limitations of AI is crucial to ensure that patients understand the purpose and potential outcomes of AI-assisted healthcare.

For peer review only - http://bmjopen.bmj.com/site/about/guidelines.xhtml

Strengths:

- The study's strength lies in interviewing women actively undergoing mammography with AI as a third reviewer, addressing hypothetical bias.
- This study provides valuable input for future ethical and legal considerations in AI integration in healthcare.

Limitations:

- Findings may be specific to the AI tool at Capio S:t Görans Hospital with Philips equipment, limiting transferability to other AI tools.
- The study primarily included participants with high trust in healthcare, potentially biasing results toward positive attitudes about AI implementation.

For peer review only

For peer review only - http://bmjopen.bmj.com/site/about/guidelines.xhtml

Introduction

Population-based breast cancer screening programs face the challenge of a heavy workload for radiologists assessing the breast X-ray images of mostly healthy women. Another challenge is the proportion of clinically detected breast cancers during the time between two consecutive screening examinations. These cancers, known as interval cancers, are associated with increased rates of both mortality and morbidity [1]. There are several ways in which artificial intelligence software (AI) could improve the screening process. An AI cancer detection algorithm could work as a concurrent assistant to a radiologist and find additional cancers. It can also be used as an independent reader of the mammograms to reduce the workload for the radiologists as well as help in triaging patients in first-line care [2].

AI systems have been introduced in some hospitals and clinics in Sweden, and their use is expected to increase dramatically in the coming years [3, 4]. Retrospective studies have demonstrated that an AI cancer detection algorithm could perform on par with, or even better than, radiologists [2]. In recent years, there has been great progress in terms of improving AI accuracy as well as evaluating it as a tool, and several tumor detection algorithms have been developed for mammography [5-7]. Retrospective studies have shown that standalone AI could assess mammograms with equal accuracy as radiologists [8]. Two recent prospective clinical studies have confirmed that AI can be integrated into the screening workflow and help radiologists detect more cases of cancer at a lower cost [9, 10]. However, challenges such as overdiagnosis remain, which should be considered in further development and implementation [11].

Dembrower [9] performed a prospective clinical trial following a paired screen-positive study design. In total, 55,581 women were included between April 2021 and June 2022. The aim of that study was to assess whether one reader in a double reading setting for screening mammograms could be replaced by an AI algorithm (AI tool with Philips equipment). The

For peer review only - http://bmjopen.bmj.com/site/about/guidelines.xhtml

study determined that such an algorithm led to increased cancer detection and a lower recall rate than standard-of-care double reading by two radiologists. We've built upon this study to explore women's perceptions, based on the idea that their direct experiences in this specific context yield more authentic insights.

A high attendance rate is important for population-based screening programs to reach more women. The breast screening program in Sweden has high participation, with approximately 75%. High participation rates are important, but also crucial is ensuring this comes from women's informed choices and trust in the AI's reliability and the program's integrity. Therefore, women attending the screening program are important stakeholders in the implementation of AI. To ensure women's trust in the screening program and survival rates, it is crucial to address practical aspects when considering the implementation of AI systems in medical imaging [12]. Understanding women's views is essential for an AI implementation that is not only efficient but also respectful of their informed decision-making rights.

Assessing the attitudes of the public, patients, and healthcare personnel toward the use of AI in healthcare is a topic of great interest [13]. Studies suggest a positive outlook on AI in healthcare, yet emphasize the need for human involvement in decision-making, highlighting the importance of a balanced, respectful approach [14], and appreciate human interaction in the diagnostic process [15, 16] [17]. Some studies also indicate that women want to be fully informed about the use of AI in mammography [18].

This study aimed to explore perceptions and attitudes toward the use of AI in the national Swedish breast cancer screening program among women participating in the national breast cancer screening program.

For peer review only

Methods

This prospective study was approved by the Swedish Ethical Review Authority (Dnr 2022-04968-01) and performed at Capio S:t Görans Hospital in Stockholm. All participants provided oral and written consent. This article is a component of the AICare research project, which seeks to identify and anticipate legal challenges, while also proposing measures to ensure patient safety and enhance public acceptance of AI in healthcare. Conducted as a qualitative, semi-structured interview study, this article will contribute to informing future legal and ethical analyses in this field.

The setting and recruitment

Every day, 350 women, between 40 and 74, undergo their biannual screening examination at the hospital. Participation in the screening program is voluntary and free-of-charge. The participants can reschedule their appointment online or via telephone if necessary. Upon arrival, the participants register their presence using their social security numbers and proceed to the waiting area where they await their turn. Subsequently, radiographers help the women throughout the screening examination, which generates four images, with two views of each breast. They also ask the women questions about clinical symptoms from their breasts, such as new lump(s), secretion, eczema, and skin retraction. The screening examination lasts approximately five minutes. If there are no clinical findings and the mammograms are assessed with no suspicion of cancer, the women receive their mammography results by mail after approximately two weeks.

In the prospective clinical study, ScreenTrustCAD, the results of adding an AI algorithm with Philips equipment to the mammography reading at Capio S:t Göran hospital, was studied. If any reader, AI or radiologists flagged an examination for a suspicious image finding, the examination was discussed at a special meeting called a consensus discussion. On this

For peer review only - http://bmjopen.bmj.com/site/about/guidelines.xhtml

occasion, at least two experienced breast radiologists assessed the mammograms and together decided whether the woman should be declared healthy or recalled for further follow-up. The current interview-based study was conducted at the same time as the ScreenTrustCAD study, i.e., AI was already being used and informed for the women in this study.

Participants (i.e., women participating in mammogram screening) were recruited at the mammography department at Capio S:t Göran Hospital, Sweden. In total, 63 women were invited by the first author (JVJ) to participate in the study before or after the screening procedure. All women were informed in the waiting room and in the consultation room that they were participating in a clinical study. Sixteen women consented, and two of them agreed to attend an interview via Zoom due to a lack of time in conjunction with their screening appointment.

The participants

The participants were very positive about the breast cancer screening program. The majority of the women had never been recalled. Those women who had reported a lump or been recalled expressed that it was very stressful. None of the women had been previously diagnosed and treated for breast cancer. Additional characteristics of the participants are presented in Table 1.

For peer review only

Table 1. The characteristics of the participants (N=16) are presented as frequencies and percentages.

**Questions**	n (%)
**What is your age?**	
41–50	5 (31.3)
51–60	6 (37.5)
61–70	5 (31.3)
70+	0 (0)
**What is your highest level of education?**	
Less than secondary/high school	1 (6.3)
Secondary/high school	4 (25.0)
University	11 (68.8)
**Have you been called back for a follow-up of your mammogram?**	
Never	13 (81.3)
Once	3 (18.8)
**Compared to other individuals in Sweden of the same age, how do you assess your risk of developing breast cancer in the next 10 years?**	
Slightly lower	1 (6.3)
Neither higher nor lower risk	7 (43.8)
Slightly higher	1 (6.3)
Much higher risk	2 (12.5)
Do not know/have no idea about it	5 (31.3)
**Are you worried about developing breast cancer?**	
Not worried at all	3 (18.8)
A little worried	12 (75.0)
Very worried	1 (6.3)
**Has anyone in your immediate family been affected by breast cancer? (grandmother, aunt, mother, or sister)**	
Yes	2 (12.5)
No	12 (75.0)

Do not know	2 (12.5)

Data collection

The interviews were performed in February 2023 by the first author (JVJ). They lasted from 15 to 36 minutes each, and they were conducted in Swedish, except one that was conducted in English. We began every interview by asking the participant about her experience of undergoing mammography. We introduced AI in a general context, inquiring about its capabilities. Thereafter, we delineated its application in mammography, illustrating how the AI, a computer program, scans mammogram images and detects irregularities, prompting a response if any deviation from healthy mammograms is detected. Thereafter, we asked about the women's knowledge, thoughts, perceptions and attitudes of the technology in general, especially for evaluating mammograms. In this study, 'perception' refers to participants' individual understanding and viewpoints on AI in breast screening, while 'attitude' encompasses their emotional and evaluative responses towards its use. See Table 2 for the semi-structured interview guide with open-ended questions [19, 20].

For peer review only

For peer review only - http://bmjopen.bmj.com/site/about/guidelines.xhtml

Table 2. The interview guide used for the semi-structured interviews primarily focused on open-ended questions to explore the women's perceptions of AI in evaluating their mammograms, although some follow-up questions were more direct to encourage deeper discussion and insight.

**1. Can you start by telling me about your experience undergoing mammography screening?**	
**2. Description of how the mammogram images are assessed and the clinical trial with AI.**	
	What do you think about that? How does it feel? To what extent do you perceive the assessment process as reliable?
	What do you think about AI in the assessment? What emotions does this information evoke?
	Do you feel okay with a doctor being replaced by AI? Could the involvement of AI change your trust in the assessment process? In what way?
	If we imagine that AI would replace both radiologists in the assessment, then you will not receive any other assessment by a doctor. Do you think that is okay (i.e., no human involvement)?
**3. Description of AI and radiologist's work and missing cancer.**	
	What do you think about this? How do you feel about this?
	Would it be different if a doctor or AI misses seeing signs of cancer in the images? If the AI misses it, whose responsibility do you think it is? How do you feel about this?
	It is possible to set AI to signal for everything, even the smallest change, which would make doctors assess many images to determine if a woman should be contacted. As a result, many women will receive a letter stating that they need further tests to rule out cancer, even though not all women will have cancer. What do you think about that? Does it worry you?
	If AI is set to be less sensitive, fewer women will receive such a letter, reducing unnecessary worry. However, this would increase the risk of some women having undetected cancer. On the other hand, healthcare resources would be focused on more severe cases of cancer. What do you think about that? Does it worry you?
	In the long run, imagine AI replacing both radiologists to assess the images. What do you think about that? What risks or concerns do you have?
**4. Description of the development of healthcare through the sharing of healthcare data.**	
	What information about yourself do you consider sensitive? What is acceptable for you to share within healthcare, and what is more sensitive?

For peer review only

	Is a mammogram image (show the image) sensitive if it were to be shared with other hospitals or companies developing these algorithms?
	How do you feel if no other information about you could be linked to your image, only the result of the examination (cancer or not)? Would that be sensitive for you?
**5. Closing questions**	
	After discussing your perspective on using AI in mammography screening, do you have any final thoughts?
	Is there anything else we haven't talked about that you would like to mention?

Analysis

The recorded interviews were transcribed verbatim by a professional transcription company. Subsequently, the audio files were listened to in their entirety to verify the transcriptions. After all the transcripts were read again, meaning units (phrases, sentences, or paragraphs) related to the aim were identified for further scrutiny. The data management and analysis process was facilitated by using Atlas.ti Web [21] Microsoft Excel (2016) as a tool. The next phase of the process entailed comparisons of the meaning units, examining their similarities and differences from the perspective of perception of AI in mammography. Open coding of each meaning unit was added. Two of the interviews were coded simultaneously by both authors (JVJ and ÅG), who jointly discussed what meaning units to identify, interpretations, and formulations of codes; they also created coding frameworks to improve conceptual clarity or transparency in the data analysis. The remaining interviews were coded by JVJ, but regular follow-up meetings were held to discuss new codes. Codes that reflected a similar concept were grouped; sub-categories were formulated, and categories were identified [22, 23] by JVJ and thereafter discussed thoroughly with the other authors (ÅG, FS, and KD); see Table 3. Thematic saturation was attained in alignment with the intended objectives of the data collection [24].

For peer review only - http://bmjopen.bmj.com/site/about/guidelines.xhtml

170 Table 3. An example of the analytical process regarding the perception of using AI as a decision tool evolved
171 from identifying "meaning units" and performing "initial coding" to organizing codes into "sub-categories" and,
172 ultimately, formulating overarching "categories" for a more comprehensive qualitative analysis.

**Meaning unit**	**Initial coding**	**Sub-category**	**Category**
"I think that a human being can... can see, like, 'Oh, is there something there, then maybe I have to look there, and then I have to look there, and then I have to look there.' Some kind of reasoning ability."	Humans can see connections; it's like detective work	AI is merely a tool	Perceived differences between AI and human assessment
"If it is used as a complement, I think it is good."	AI should be used as a complement	Human in the loop	Requirements when using AI in mammography
"...anonymization is important, ensuring that the AI company or any other party does not have access to personal data or information about individuals. It's crucial to protect privacy and prevent sensitive data from being linked to specific individuals."	Consider sharing deidentified mammography images with AI companies	Trust in data sharing varies in routine care, research, and industry	Trust when implementing AI in mammography

173

174 Patient and public involvement

175 None.

176

177

178

For peer review only - http://bmjopen.bmj.com/site/about/guidelines.xhtml

Results

The results below describe the perceptions and attitudes expressed by the participants regarding AI in healthcare. We identified 318 meaning units in total. When analyzing them, seven sub-categories emerged, which were categorized into three main categories: perceived differences between AI and human assessment, trust when implementing AI in mammography, and requirements when using AI in mammography. An overview of these sub-categories and main categories is presented in Table 4. These will be described and illustrated using quotes in the following sections.

Table 4. The categories and sub-categories regarding participants' perceptions of taking part in triage for mammography using AI.

Category	Sub-category
Perceived differences between AI and human assessment	AI is merely a tool
	The difference in error tolerance
Attitudes when implementing AI in mammography	Trust in the healthcare system gives confidence in AI
	Willingness to data sharing varies in routine care, research, and industry
Requirements when using AI in mammography	Evaluation of AI
	Human in the loop
	The trade-off between being worried about possible cancer and detecting cancer

For peer review only

Category: Perceived differences between AI and human assessment

Within this category, the participants talked about the positive and negative characteristics of AI and human assessment. They also talked about how their moral expectations differed between AI and humans regarding error tolerance.

Sub-category: AI is merely a tool

The participants were familiar with AI to varying degrees, primarily through their interactions with search engines and recommendation systems on the internet. In addition to the positive aspects, they had heard stories about AI being used to cheat when writing papers and exams. Some participants expressed excitement about the development of AI, recognizing its potential benefits. However, one participant cautioned against the excessive hype, stating that AI should not be equated with the futuristic concept portrayed by some. Despite the generally positive sentiment toward the utilization of AI in mammography, a subset of participants voiced reservations regarding their limited understanding of the underlying mechanisms of AI. They expressed a sense of ambivalence toward forming a definitive opinion on the benefits or drawbacks of AI implementation in mammography. After a brief presentation on how AI works at the clinic, they were all capable of expressing their attitudes toward AI. They made clear distinctions between the characteristics and abilities of an AI compared with a human; they often drew upon what humans are capable of when describing what AI can or cannot do.

> *No, but I mean, can you build a machine that is that skilled? That's what I'm thinking. In addition, the human eye and experience of how things can look; I don't think you can just cram all of that into a robot, you know.* (Participant 7)

The women acknowledged and appreciated that AI has the potential to identify patterns that may elude the human eye and respond to deviations from the norm. They perceived that AI could be a powerful tool, helping radiologists complement their work. Some expected that the use of AI would save resources for the healthcare system and give radiologists the possibility of performing other tasks. Some perceived that the AI to a great extent would complement the radiologists. They believed that AI is self-learning, quick, and will never get tired. Some

For peer review only - http://bmjopen.bmj.com/site/about/guidelines.xhtml

participants held the belief that AI technology will continue to evolve over time, surpassing its current capabilities and becoming even more advanced. They expressed that the utilization of AI represents the future of healthcare. Consequently, humans and AI were perceived as complementary entities, working together synergistically.

Nevertheless, a prevailing skepticism regarding the current capabilities of AI was evident among many participants. One expressed a general cautious perspective toward technology, acknowledging its benefits as long as it functions properly. However, some raised concerns about the sustainability of AI effectiveness in the long run. Some participants expressed uncertainty regarding AI's ability to detect all cancers, stating that AI might have difficulty with certain more uncommon conditions, such as breast implants or dense breast tissue. They also doubted whether AI could be good enough to replace the two radiologists currently assessing the mammography images. On the other hand, AI could be good at detecting things that the human eye cannot. However, participants expressed uncertainty regarding the specific areas in which AI excels beyond the capabilities of the human brain.

> *I also think that a human has the ability to... some form of consequential thinking. I mean, a reasoning ability that AI possibly doesn't have.*
> (Participant 6)

Some of the major strengths attributed to humans included their holistic perspective, considering various factors related to the individual woman, and making connections between different pieces of information. Humans were also perceived as capable of thinking about consequences, conducting investigative work, and demonstrating greater imagination.

Overall, human abilities were perceived in a more positive sense compared to AI. Many participants, however, expressed a willingness to accept the use of AI if it could outperform radiologists in terms of detecting more cases of cancer. The primary concern for them was the timely and accurate identification of cancer, regardless of the method used. However, they

For peer review only

also strongly believed that human involvement adds an irreplaceable element that AI currently lacks and perhaps never will possess. Participants emphasized that humans bring unique qualities and abilities to the screening process that go beyond mere detection, such as intuition, empathy, and contextual understanding. While they acknowledged the potential of AI, they maintained that human touch and contribution are invaluable and offer something distinct that AI cannot yet replicate.

> *I'm not a hundred percent convinced that the human eye can be replaced because there are... we also had scanning microscopes and things like that. That was a hundred years ago now... but, I mean, it [AI] is an aid.* (Participant 3)

Sub-category: The difference in error tolerance

The ability of AI to detect cancer in mammography was consistently emphasized by all participants, and the failure to detect cancer was perceived as devastating. However, their acceptance of failure to detect cancer differed between radiologists and AIs, having a much higher acceptance for mistakes made by radiologists than by an AI. This was because participants felt that to err is human – and therefore understandable. In contrast, the majority expected that technology, including AI, should not make mistakes when used in healthcare. They believed that AI, to be acceptable upon implementation, should function well, maintain a high level of security, and operate without errors. They questioned the rationale behind implementing AI if it did not meet these criteria. Two participants specifically highlighted the potential consequences of AI-making mistakes that could be catastrophic due to the systematic nature of errors. In contrast, they pointed out that if a single radiologist were to miss a few cases of cancer in a day, the impact would be comparatively limited. The participants expressed a range of negative emotions, including feeling bad, disappointment, and cheating if AI was to make a mistake. One participant held a pessimistic view of

For peer review only - http://bmjopen.bmj.com/site/about/guidelines.xhtml

technology and questioned whether it could guarantee reliable performance at all times, despite acknowledging its current functionality.

> *Yes, because we all know that a technology […] it should be a hundred percent...* (Participant 2)

A few participants expressed a more nuanced perspective, acknowledging that neither humans nor AI can be expected to be 100% accurate all the time. They recognized that AI relies on data and statistical predictions, which inherently introduces the possibility of errors. These participants emphasized that no system, whether human or AI, is infallible, and both can potentially make mistakes in their assessments.

The participants held high expectations of the radiologists and regarded them as having ultimate responsibility for the final results. Additionally, some participants believed that if AI were to make a mistake, it would be the responsibility of humans, as AI is viewed as a tool incapable of being held accountable for its decisions.

> *[…] It is always the medically responsible physician, the radiologist, who is accountable. We can't blame the technology. We must have technology that is tested and that we believe in and trust. It's always the medically responsible physician, in my opinion. Where else should the responsibility lie, I wonder. That's how I think.* (Participant 4)

However, one respondent expressed an opposing viewpoint, suggesting that if a radiologist were to make a mistake, it could be attributed to a flaw in the device or image-computer system. Another participant highlighted the importance of the radiologist not disregarding the indications provided by AI when it signals a case of potential cancer. Overall, the participants' perceptions varied regarding the issue of responsibility.

For peer review only

For peer review only - http://bmjopen.bmj.com/site/about/guidelines.xhtml

Category: Attitudes when implementing AI in mammography

This category and its sub-categories concerned women's attitudes in sharing mammography images with different actors, as well as trust in the healthcare system's implementation of AI. Here, we identified two sub-categories.

Sub-category: Trust in the healthcare system gives confidence in AI

This sub-category predominantly reflects the participants' strong trust in the healthcare system in regard to implementing AI in routine practices. All the participants expressed a high level of confidence that healthcare providers would take the necessary steps to deliver the best possible care, even if it involved the implementation of AI.

> *I trust that hospitals do it to the best of their ability.* (Participant 6)

While some participants acknowledged the potential for unwanted motivations, such as cost-saving measures behind AI implementation, they still expressed trust that both healthcare providers and developers of AI tools have the intention of saving lives and improving patient outcomes. Some participants even indicated that if healthcare professionals have good intentions in utilizing AI, they do not feel the need to have detailed information regarding its use.

> *No, I think experts must have examined and evaluated that, so no, I would probably trust... I mean, I think if they have chosen it, it is because it's very effective and accurate.* (Participant 9)

Sub-category: Willingness to data sharing varies in routine care, research, and industry

The participants consistently expressed their willingness to share their health information within the healthcare system setting. Furthermore, many participants held a strong belief that different healthcare units should collaborate and share information more extensively than they currently do, emphasizing that such sharing is crucial to ensure patient safety. However,

For peer review only - http://bmjopen.bmj.com/site/about/guidelines.xhtml
For peer review only

in regard to sharing health-related data or personal data with companies, the participants were more reluctant. For some, it was perceived as a great violation of their privacy. The participants felt that mammogram images of their breasts were less controversial if they were completely anonymized and not connected to them as individuals. Anonymization of the mammograms helped to alleviate concerns regarding privacy and personal identification.

> *No, I wouldn't have any issues with that [sharing mammograms] as long as it can't be linked to me as a specific individual.* (Participant 6)

However, for many of the participants, providing consent to share the data were viewed as necessary and connected to their ability to influence that the mammograms would be used for a meaningful purpose.

> *On the other hand, if it's a research project or a pilot project, then you want to know that you're participating in such a project. However, if it's part of the established methodology at hospitals... I don't interfere with that.* (Participant 6)

> *I would like to know beforehand so that they don't just send it off without informing me. It doesn't feel entirely right. I would expect to be informed about it, though.* (Participant 8)

They were willing to consent if the purpose was to save lives, develop new medical devices, or conduct research. Conversely, if the data usage was intended for commercial companies or advertising purposes, they expressed a strong reluctance to share their data.

Category: Requirements when using AI in mammography

This category reflects the prerequisites expressed by our participants regarding the use of AI in the mammography screening process.

For peer review only

Sub-category: Evaluation of AI

This sub-category related to the paramount evaluation of AI in multiple aspects. Participants highlighted the need to evaluate AI's cancer detection performance compared to radiologists and the overall integration process and integration process. Participants stressed evaluating professionals' perspectives, AI image selection criteria, and the impact on radiologists' skills. They underscored the importance of radiologists' continuous training, decision-makers' understanding of AI's implications, and the need for control and monitoring to ensure AI's accuracy and relevance to the Swedish context.

The participants expressed concern that decision-makers might focus solely on certain aspects, such as cost savings, when evaluating AI, potentially overlooking other important factors, such as providing comprehensive training for specialists.

The participants agreed that the evaluation of the AI's performance itself needs to focus on the ability of AI to keep the margin of error down and evaluate to what extent it performs better.

> *I think it's possible to evaluate the error margins and compare the percentage of correct or incorrect findings between the two. Therefore, I believe that would be beneficial.* (Participant 5)

Sub-category: Human in the loop

All participants preferred humans over AI; however, they expressed that combining AI and humans would be a complement to each other. The participants emphasized the need for human oversight due to their perception of the unique qualities possessed by radiologists and humans; even though AI is a powerful tool, it requires human control. They expressed the desire to harness the combined strengths of both humans and AI. Moreover, participants believed that a collaborative approach, where AI assists radiologists in detecting abnormalities, would yield the best outcomes.

For peer review only - http://bmjopen.bmj.com/site/about/guidelines.xhtml

> *No, but I think... as long as there is a human factor involved, I believe it can be very effective.* (Participant 6)

Some of the participants identified the risk that using AI too much would lead to a decline in learning skill development for the radiologist. They reasoned that if AI gives too many false positives, it will create a misleading foundation for radiologists to identify cancer cases. They expressed concerns that if AI solely performed the initial assessment, radiologists would only discuss a selected sample of images identified by AI. One respondent had a different perspective and was less concerned about radiologists losing competence. Instead, she understood that the development of AI requires radiologists to continuously improve their skills. She recognized that to effectively contribute to the development and training of AI systems, radiologists must stay updated and enhance their expertise. This viewpoint highlights the symbiotic relationship between radiologists and AI, where ongoing skill development and collaboration contribute to the advancement of both fields.

> *But it is still the case that radiologists constantly need to enhance their competence to develop AI. Because it's the people who collect the data; it's not like an external force that does it.* (Participant 4)

Some expressed a hopeful perspective regarding the combination of AI and human involvement in preventing potential errors. They perceived that there is too much uncertainty regarding the performance of AI, which led them to believe that relying solely on AI at the present moment would be a substantial leap. They expressed fears that something will be lost and that the radiologists will undermine their role in the diagnostic process; AI can serve as a support tool, but a human needs to be in control. All participants thought it was acceptable to implement AI as long as one radiologist was still involved in the first round. However, one participant expressed that it was compulsory to like digitization, as it is a reflection of our society's direction. She expressed a preference for a collaborative approach, desiring the

For peer review only

presence of a radiologist in the process and appreciation for human contact and interaction in the process.

Sub-category: The trade-off between being worried about possible cancer and detecting cancer

The participants strongly expressed that failing to detect a case of cancer is terrible, and they emphasized the importance of healthcare providers doing everything possible to ensure early detection. They recognized the trade-off between causing increased worry among women due to more false positive cases and the potential risk of missing a cancer diagnosis after explaining that this was the reality. However, they expressed a clear preference for prioritizing the detection of potential cancer cases over minimizing false positives. They believed that it was better for more individuals to experience the anxiety of false-positive results than for a potential case of cancer to go undetected.

> *I think we should be a bit more... we should be able to live a bit more in reality [not being afraid of bad news]. And if we can get the help [hinder cancer] that it entails, that I have to go for an extra appointment, then I would just be thankful and accept it. I'm not inclined in the other direction. No, I would rather go for the extra appointment than not do it.* (Participant 4)

Participants agreed on setting AI to a highly sensitive level for better cancer detection and emphasized the need for transparent communication about AI use in Swedish healthcare. They believed women would accept AI if it improved cancer detection and advocated for detailed information in referral letters about potential increased callbacks due to AI's sensitivity.

For peer review only - http://bmjopen.bmj.com/site/about/guidelines.xhtml

> Then, they must communicate that 'the experience has shown that about thirty percent of those who have this develop cancer, or zero or twenty or forty.' Something like that. I think it's good to communicate that, if you ask me. *(Participant 1)*

Participants preferred immediate follow-ups to lessen anxiety, viewing screening as an act of solidarity and accepting false positives for timely cancer treatment for others. They saw the initial resource investment in AI as ultimately cost-saving through early detection. Emphasizing transparency to maintain trust, they indicated they wouldn't opt out of mammography with AI integration, underscoring the importance of open communication in healthcare AI.

For peer review only - http://bmjopen.bmj.com/site/about/guidelines.xhtml

Discussion

The main findings

The main findings of this study demonstrate that participants perceive AI as a valuable tool in mammography, recognizing the potential benefits and its ability to complement the work performed by radiologists. However, there are concerns about participants' limited understanding of AI and reservations about its current capabilities. Participants stressed human involvement, transparent screening, and evaluating AI's ability to detect cancer. They prioritized finding potential cancers over reducing false positives and believed that women would accept AI if it detected more cases. The emphasis on cancer detection, while crucial, poses challenges due to limited healthcare resources. Lowering sensitivity thresholds raises ethical questions about costs and personnel. It is imperative to explore the lessons learned from Sweden's mammography program and consider the ethical, defensible trade-offs, especially in resource allocation. Striking a balance between optimal detection and efficient resource use remains a pivotal area for healthcare policy discussions.

The results underscore the importance of a collaborative approach, where AI and radiologists complement each other and where radiologists maintain a vital role in the diagnostic process by contributing with their "human capabilities." These findings align with other patient groups, where participants emphasized the need for, and value of, information, even if it is not used in medical decision-making [25]. The study shows that it is crucial to inform patients about the use of AI as a straightforward clinical implication, aiming to maintain participants' trust. Additionally, the study highlights that human involvement in medical care is highly valued, and it would be difficult to replace it with AI. This is a consistent theme identified in studies outside the realm of mammography [26] [27].

For peer review only

Discussion of each finding

Within the first category in our study, we discovered perceptions regarding differences between AI and human assessment. Participants were cautiously optimistic about AI in healthcare, acknowledging its benefits but expressing reservations due to current limitations in detecting all cancers and conditions. Interestingly, these views align with those of participants in prior studies where concerns about AI replacing radiologists entirely, apprehensions regarding the absence of a ‘human touch’ in the diagnostic process [14, 17], and accountability issues hindered the acceptance of AI-driven medicine [28, 29]. Moreover, our study emphasizes the importance of retaining human expertise and clear lines of responsibility in breast cancer screening. Participants underlined these aspects, emphasizing the need for a specific framework, although such guidelines are still under development. The National Board of Health and Welfare in Sweden has initiated work on guidelines for AI in healthcare,[30] a step in the right direction. This emphasis aligns with prior research stressing the importance of preserving human involvement in breast screening [31, 32]. It highlights the complexity of AI perception in healthcare, emphasizing the necessity for tailored guidelines that address regulatory and ethical concerns while catering to the specific demands of distinct medical fields.

It is particularly intriguing to note that participants in our study placed significant emphasis on the consequences of technological errors. Most women held high expectations for AI, anticipating its flawless performance, unwavering security, and a complete absence of errors. Strikingly, when contemplating the prospect of AI making mistakes, participants expressed strong negative emotions. This contrasts with the perceptions of healthcare professionals, where a certain degree of error was deemed more acceptable, attributed to the inherent human factor [33, 34]. People in general tolerate fewer mistakes made by technical products than mistakes made by humans. A pivotal observation was the varying perspectives on responsibility among the participants. While many ascribed the ultimate responsibility for

potential errors to radiologists, a subset viewed AI as an entity lacking in accountability. These attitudes describe the intricate interplay between human trust, the technology's reliability, and the attribution of responsibility in the context of AI implementation within healthcare. This divergence in tolerance towards errors by humans versus AI underscores a psychological aspect of trust in healthcare. Participants view human errors as part of learning, but see AI mistakes as systemic flaws, challenging trust in technology. This dichotomy raises critical questions about how we educate and prepare the public for the realistic capabilities and limitations of AI in healthcare, balancing technological optimism with a pragmatic understanding of AI as an evolving tool rather than an infallible solution.

In our study, the category "Attitudes when Implementing AI in Mammography" revealed that participants generally exhibited trust in the integration of AI into routine mammography practices. They believed that healthcare providers and AI developers shared a common goal of saving lives and enhancing patient outcomes. However, willingness had boundaries, as participants were reluctant to share health information with external companies, considering it an intrusion into their private lives.

The participants expressed that maintaining the confidentiality of the mammogram images played a pivotal role in addressing privacy concerns, making participants more willing to share their data as long as it could not be traced back to them as individuals. Consent for data sharing was often contingent on meaningful purposes, such as life-saving measures or research initiatives. Conversely, participants expressed hesitation about sharing data for commercial or advertising purposes. This aligns with previous studies on people's attitudes toward data sharing [35]. There is a risk that the participants do not comprehend the necessity of sharing data with private entities to advance technology.

In summary, our study underscores the pivotal role of attitudes in AI implementation in healthcare. Trust, rooted in transparent collaboration between healthcare providers and participants, is fundamental. Recognizing its importance is crucial in the complex realm of AI

integration. Collaborative efforts, informed by human rights frameworks and practical recommendations, are essential. These actions not only preserve but also nurture trust, ensuring the inclusive and effective application of AI technology in healthcare [36, 37].

In the third category, participants expressed their thoughts on prerequisites for the implementation of AI in mammography screening. In line with these expressed prerequisites, it is evident that the implementation of AI in healthcare, particularly in mammography screening, has brought to the forefront a multitude of ethical considerations. These considerations closely align with the broader discussions in the literature surrounding the ethical dimensions of AI integration [11, 38]. These include evaluating AI's performance compared with that of radiologists, ensuring transparency in the screening process, and addressing concerns about the potential loss of expertise among radiologists. The participants stressed the importance of ongoing training for radiologists and urged decision-makers to consider factors beyond cost savings. It is therefore important that there is a solution for unexperienced radiologists to undergo training in mammography screening. Trust in AI is expected to increase as familiarity with its capabilities grows. This was also observed among the radiologists when the ScreenTrust CAD study started, as recall rates increased during the first few months and later normalized. Although participants in that study preferred human involvement in the screening process, they recognized the potential of AI in detecting abnormalities and believed that a combination of both would yield the best outcomes, which the ScreenTrust CAD study confirmed [9]. Participants prioritized the early detection of cancer, even if it meant accepting more false-positive results and having to return for further follow-up examinations. Participants saw the implementation of AI as initially requiring additional resources but ultimately saving money by detecting cancers earlier with fewer resources.

Strengths and limitations

This study, focusing on women's perceptions and attitudes toward AI in mammography, offers insights for shaping ethical frameworks and legal guidelines in AI healthcare applications. While not a bioethical analysis per se, it provides valuable input for future ethical and legal considerations in AI integration in healthcare. However, the findings may only be applicable to this AI tool at Capio S:t Görans Hospital with Philips equipment. The study excluded other AI-developed tools, which may limit the transferability of the results. One major strength of our study is that we interviewed women who were actively undergoing mammography, where AI was utilized as a third reviewer. This means that we have addressed the hypothetical bias commonly encountered in studies [39].

There are potential limitations in self-reporting bias and subjectivity in interview-based studies. To address these limitations, the researchers encouraged honest and candid responses, maintained confidentiality, reflected on their own perspectives and biases, sought input from colleagues, and used coding frameworks to improve objectivity in data analysis [40]. Considering transferability, we identify a limitation: our study predominantly intercepted participants who exhibited high trust in healthcare, a sentiment likely to influence their confidence in the implementation of AI. To address this gap, we recommend examining specific demographic groups to gain comprehensive insights. Some people with low trust in conventional healthcare might also be skeptical about AI, while others might perceive AI as more objective, leading to higher trust. This viewpoint is supported by Yang et al.,[26] indicating varied levels of trust based on demographics. Understanding these dynamics is vital to grasp societal attitudes toward AI in healthcare.

For peer review only

Relevance

In summary, this study’s findings hold significant relevance for the ongoing debate surrounding AI in healthcare, especially within breast cancer screening programs. This highlights the essential role of trust in AI and the necessity of retaining human involvement in making decisions regarding mammogram images. These insights are vital for ensuring the effective and well-received implementation of AI, emphasizing transparent communication and comprehensive patient education regarding the role of AI in breast cancer screening. In this study, participants clearly emphasized the ethical principles of fairness, privacy, responsibility, and accuracy while underscoring the competence of radiologists and the importance of transparency. Their overall positive attitude toward the technology is evident.

For peer review only

Conclusion

The findings of this study indicate that women who participate in mammography view AI as one of many tools in the healthcare system, not as a standalone solution. They acknowledge its potential but emphasize its role in complementing existing practices rather than replacing the radiologists entirely. However, if healthcare professionals determine that AI functions equally or even better than traditional screening processes, then the participants find the implementation of AI without explicit consent acceptable. Notably, women prefer AI to be sensitive in detecting potential cancer, even if it may lead to an increased level of unnecessary worry and fear. Nevertheless, effective communication regarding the role and limitations of AI is crucial to help patients understand its purpose and potential outcomes in healthcare and to maintain their trust.

Declarations

Funding

This project was supported by WASP-HS (Grant agreement no. MMW 2020.0093, Project AICare). The funding agreement ensured the authors' independence in designing the study, interpreting the data, and writing and publishing the report.

Ethics approval and consent to participate

The study was approved by the Swedish Ethical Review Authority (Dnr 2022-04968-01). Oral and written consent to participate was obtained from all participants. The interviews were carried out according to the Declaration of Helsinki and prioritized participants' privacy and safety. Participants were informed that they could withdraw from the study at any time with no explanation and that ending would not affect the potential treatment needed. Names were replaced with codes to remove all personal identifiers.

Data sharing statement

No additional data are available.

Competing interests

None.

Contributions statement

JVJ, KD, FS and ÅG conceptualized the study. JVJ performed the interviews. JVJ and ÅG analyzed the interviews and interpreted the data. JVJ was the major contributor in writing the manuscript. All authors revised the manuscript substantively. All the authors read and approved the final manuscript.

For peer review only

References

1. Törnberg, S., et al., *A pooled analysis of interval cancer rates in six European countries.* European Journal of Cancer Prevention, 2010. **19**(2): p. 87-93.
2. Dembrower, K., et al., *Effect of artificial intelligence-based triaging of breast cancer screening mammograms on cancer detection and radiologist workload: a retrospective simulation study.* The Lancet Digital Health, 2020. **2**(9): p. e468-e474.
3. Buch, V.H., I. Ahmed, and M. Maruthappu, *Artificial intelligence in medicine: current trends and future possibilities.* 2018. **68**(668): p. 143-144.
4. Horgan, D., et al., *Artificial Intelligence: Power for Civilisation – and for Better Healthcare.* Public Health Genomics, 2019. **22**(5-6): p. 145-161.
5. Chan, H.-P., R.K. Samala, and L.M. Hadjiiski, *CAD and AI for breast cancer—recent development and challenges.* 2020. **93**(1108): p. 20190580.
6. Schaffter, T., et al., *Evaluation of Combined Artificial Intelligence and Radiologist Assessment to Interpret Screening Mammograms.* JAMA Network Open, 2020. **3**(3): p. e200265-e200265.
7. McKinney, S.M., et al., *International evaluation of an AI system for breast cancer screening.* Nature, 2020. **577**(7788): p. 89-94.
8. Yoon, J.H., et al., *Standalone AI for Breast Cancer Detection at Screening Digital Mammography and Digital Breast Tomosynthesis: A Systematic Review and Meta-Analysis.* Radiology, 2023. **307**(5): p. e222639.
9. Dembrower, K., et al., *Artificial intelligence for breast cancer detection in screening mammography in Sweden: a prospective, population-based, paired-reader, non-inferiority study.* J The Lancet Digital Health, 2023.
10. Lång, K., et al., *Artificial intelligence-supported screen reading versus standard double reading in the Mammography Screening with Artificial Intelligence trial (MASAI): a clinical safety analysis of a randomised, controlled, non-inferiority, single-blinded, screening accuracy study.* J The Lancet Oncology, 2023. **24**(8): p. 936-944.
11. Carter, S.M., et al., *The ethical, legal and social implications of using artificial intelligence systems in breast cancer care.* The Breast, 2020. **49**: p. 25-32.
12. Omoumi, P., et al., *To buy or not to buy—evaluating commercial AI solutions in radiology (the ECLAIR guidelines).* European Radiology, 2021. **31**(6): p. 3786-3796.
13. McCradden, M.D., T. Sarker, and P.A. Paprica, *Conditionally positive: a qualitative study of public perceptions about using health data for artificial intelligence research.* 2020. **10**(10): p. e039798.
14. Lennox-Chhugani, N., et al., *Women's attitudes to the use of AI image readers: a case study from a national breast screening programme.* BMJ health & care informatics, 2021. **28**(1): p. e100293.
15. Ongena, Y.P., et al., *Patients' views on the implementation of artificial intelligence in radiology: development and validation of a standardized questionnaire.* European Radiology, 2020. **30**(2): p. 1033-1040.
16. Haan, M., et al., *A Qualitative Study to Understand Patient Perspective on the Use of Artificial Intelligence in Radiology.* Journal of the American College of Radiology, 2019. **16**(10): p. 1416-1419.
17. Ongena, Y.P., et al., *Artificial Intelligence in Screening Mammography: A Population Survey of Women's Preferences.* Journal of the American College of Radiology, 2021. **18**(1, Part A): p. 79-86.
18. Ploug, T. and S. Holm, *The right to refuse diagnostics and treatment planning by artificial intelligence.* Medicine, Health Care and Philosophy, 2020. **23**(1): p. 107-114.

For peer review only - http://bmjopen.bmj.com/site/about/guidelines.xhtml

19. Kvale, S. and S. Brinkmann, *Interviews: Learning the craft of qualitative research interviewing*. 2009: Sage.
20. Krueger, R.A. and M.A. Casey, *Focus groups: A practical guide for applied research*. 2014: Sage Publications.
21. ATLAS.ti Scientific Software Development GmbH. *ATLAS.ti Web - The easiest way to uncover qualitative insights*. 2022 [cited 2022; Available from: https://atlasti.com/atlas-ti-web.
22. Burnard, P., *Writing a qualitative research report.* Nurse Education Today, 2004. **24**(3): p. 174-179.
23. Burnard, P., et al., *Analysing and presenting qualitative data.* British Dental Journal, 2008. **204**(8): p. 429-432.
24. Malterud, K., V.D. Siersma, and A.D. Guassora, *Sample size in qualitative interview studies: guided by information power.* J Qualitative health research, 2016. **26**(13): p. 1753-1760.
25. Grauman, Å., et al., *Personalizing precision medicine: Patients with AML perceptions about treatment decisions.* Patient Education and Counseling, 2023. **115**: p. 107883.
26. Yang, K., et al., *Attitudes Of Chinese Cancer Patients Toward The Clinical Use Of Artificial Intelligence.* Patient Prefer Adherence, 2019. **13**: p. 1867-1875.
27. Juravle, G., et al., *Trust in artificial intelligence for medical diagnoses.* Prog Brain Res, 2020. **253**: p. 263-282.
28. Tamori, H., et al., *Acceptance of the use of artificial intelligence in medicine among Japan's doctors and the public: a questionnaire survey.* J JMIR human factors, 2022. **9**(1): p. e24680.
29. Pesapane, F., et al., *Women's perceptions and attitudes to the use of AI in breast cancer screening: A survey in a cancer referral centre.* J The British Journal of Radiology, 2023. **95**(1141): p. 20220569.
30. Österberg, M. and L. Lindsköld. *AI for better health*. 2018; Available from: https://swelife.se/wp-content/uploads/2021/01/AI_for_better_health_final.pdf.
31. Carter, S.M., et al., *Australian women's judgements about using artificial intelligence to read mammograms in breast cancer screening.* 2023. **9**: p. 20552076231191057.
32. Jonmarker, O., et al., *The future of breast cancer screening: what do participants in a breast cancer screening program think about automation using artificial intelligence?* Acta Radiologica Open, 2019. **8**(12): p. 2058460119880315.
33. Hendrix, N., et al., *Artificial intelligence in breast cancer screening: primary care provider preferences.* Journal of the American Medical Informatics Association, 2020. **28**(6): p. 1117-1124.
34. Zhang, Z., et al., *Patients' perceptions of using artificial intelligence (AI)-based technology to comprehend radiology imaging data.* J Health Informatics Journal, 2021. **27**(2): p. 14604582211011215.
35. Viberg Johansson, J., et al., *Publics' preferences for sharing health data: a discrete choice experiment.* JMIR Medical Informatics 2021. **in press**.
36. Gibbons, E.D. and S. Magazine, *Toward a more equal world: the human rights approach to extending the benefits of artificial intelligence.* J IEEE Technology, 2021. **40**(1): p. 25-30.
37. Secinaro, S., et al., *The role of artificial intelligence in healthcare: a structured literature review.* J BMC medical informatics, 2021. **21**: p. 1-23.
38. Murphy, K., et al., *Artificial intelligence for good health: a scoping review of the ethics literature.* BMC Medical Ethics, 2021. **22**(1): p. 14.
39. Loomis, J.J.J.o.E.S., *What's to know about hypothetical bias in stated preference valuation studies?* 2011. **25**(2): p. 363-370.
40. Lincoln, Y.S. and E.G. Guba, *Naturalistic inquiry*. 1984, Beverly Hills, CA: SAGE.

For peer review only

702

For peer review only

COREQ (COnsolidated criteria for REporting Qualitative research) Checklist

A checklist of items that should be included in reports of qualitative research. You must report the page number in your manuscript where you consider each of the items listed in this checklist. If you have not included this information, either revise your manuscript accordingly before submitting or note N/A.

Topic	Item No.	Guide Questions/Description	Reported on Page No.
**Domain 1: Research team and reflexivity**			
Personal characteristics			
Interviewer/facilitator	1	Which author/s conducted the interview or focus group?	7,29
Credentials	2	What were the researcher's credentials? E.g. PhD, MD	1
Occupation	3	What was their occupation at the time of the study?	1
Gender	4	Was the researcher male or female?	1
Experience and training	5	What experience or training did the researcher have?	1
Relationship with participants			
Relationship established	6	Was a relationship established prior to study commencement?	4
Participant knowledge of the interviewer	7	What did the participants know about the researcher? e.g. personal goals, reasons for doing the research	4, 5
Interviewer characteristics	8	What characteristics were reported about the inter viewer/facilitator? e.g. Bias, assumptions, reasons and interests in the research topic	6
**Domain 2: Study design**			
Theoretical framework			
Methodological orientation and Theory	9	What methodological orientation was stated to underpin the study? e.g. grounded theory, discourse analysis, ethnography, phenomenology, content analysis	7
Participant selection			
Sampling	10	How were participants selected? e.g. purposive, convenience, consecutive, snowball	4, 5
Method of approach	11	How were participants approached? e.g. face-to-face, telephone, mail, email	5
Sample size	12	How many participants were in the study?	4
Non-participation	13	How many people refused to participate or dropped out? Reasons?	4
Setting			
Setting of data collection	14	Where was the data collected? e.g. home, clinic, workplace	4
Presence of non-participants	15	Was anyone else present besides the participants and researchers?	7
Description of sample	16	What are the important characteristics of the sample? e.g. demographic data, date	6
Data collection			
Interview guide	17	Were questions, prompts, guides provided by the authors? Was it pilot tested?	8
Repeat interviews	18	Were repeat inter views carried out? If yes, how many?	no
Audio/visual recording	19	Did the research use audio or visual recording to collect the data?	9
Field notes	20	Were field notes made during and/or after the inter view or focus group?	yes
Duration	21	What was the duration of the inter views or focus group?	7
Data saturation	22	Was data saturation discussed?	9
Transcripts returned	23	Were transcripts returned to participants for comment and/or	no

For peer review only

Topic	Item No.	Guide Questions/Description	Reported on Page No.
		correction?	
**Domain 3: analysis and findings**			
Data analysis			
Number of data coders	24	How many data coders coded the data?	11
Description of the coding tree	25	Did authors provide a description of the coding tree?	10, 11
Derivation of themes	26	Were themes identified in advance or derived from the data?	9
Software	27	What software, if applicable, was used to manage the data?	9
Participant checking	28	Did participants provide feedback on the findings?	no
Reporting			
Quotations presented	29	Were participant quotations presented to illustrate the themes/findings? Was each quotation identified? e.g. participant number	11
Data and findings consistent	30	Was there consistency between the data presented and the findings?	11
Clarity of major themes	31	Were major themes clearly presented in the findings?	11
Clarity of minor themes	32	Is there a description of diverse cases or discussion of minor themes?	23

Developed from: Tong A, Sainsbury P, Craig J. Consolidated criteria for reporting qualitative research (COREQ): a 32-item checklist for interviews and focus groups. *International Journal for Quality in Health Care*. 2007. Volume 19, Number 6: pp. 349 – 357

Once you have completed this checklist, please save a copy and upload it as part of your submission. DO NOT include this checklist as part of the main manuscript document. It must be uploaded as a separate file.

For peer review only
