# Supplementary material for: Women’s perceptions and attitudes towards the use of AI in mammography in Sweden: a qualitative interview study
Source: BMJ Open. 2024 Feb 14;14(2):e084014. doi: 10.1136/bmjopen-2024-084014 (PMC10868248; doi:10.1136/bmjopen-2024-084014)
Supplement: Reviewer comments [file bmjopen-2024-084014.reviewer_comments.pdf]

Sorry, there is no peer review to display for this article.

BMJ Open operates using a fully open peer review. This means that accepted papers usually have their prepublication history posted alongside them.

This prepublication history comprises all previous versions of the manuscript, the reviewers' comments, and the authors' responses to all the comments from reviewers and editors. For clinical trials, this also includes the study protocol (submitting the protocol is mandatory for all clinical trials and encouraged for all other studies at BMJ Open).

In rare instances we determine after careful consideration that we should not make certain portions of the prepublication record publicly available. For example, in cases of stigmatised illnesses we seek to protect the confidentiality of reviewers who have these illnesses. In other instances there may be legal or regulatory considerations that make it inadvisable or impermissible to make available certain parts of the prepublication record.

In all instances in which we have determined that elements of the prepublication record should not be made publicly available, we expect that authors will respect these decisions and also will not share this information.
